# Tumor-Infiltrating Lymphocytes and Survival Outcomes in Early *ERBB2*-Positive Breast Cancer: 10-Year Analysis of the ShortHER Randomized Clinical Trial

**DOI:** 10.1001/jamaoncol.2024.6872

**Published:** 2025-02-13

**Authors:** Maria Vittoria Dieci, Giancarlo Bisagni, Stefania Bartolini, Alessio Schirone, Luigi Cavanna, Antonino Musolino, Francesco Giotta, Anita Rimanti, Ornella Garrone, Elena Bertone, Katia Cagossi, Samanta Sarti, Antonella Ferro, Federico Piacentini, Enrico Orvieto, Melinda Sanders, Federica Miglietta, Davide Massa, Sara Balduzzi, Pierfranco Conte, Roberto D’Amico, Valentina Guarneri

**Affiliations:** 1 Department of Surgery, Oncology and Gastroenterology (DiSCOG), University of Padova, Padova, Italy; 2 Oncology 2, Veneto Institute of Oncology IOV-IRCCS, Padova, Italy; 3 Department of Oncology and Advanced Technologies, Azienda USL-IRCCS, Reggio Emilia, Italy; 4 Department of Nervous System Medical Oncology, IRCCS Istituto delle Scienze Neurologiche di Bologna, Bologna, Italy; 5 S. Anna University Hospital, Ferrara, Italy; 6 Internal Medicine and Oncology, Clinica Piacenza, Piacenza, Italy; 7 Medical Oncology, Breast and GYN Unit, IRCCS Istituto Romagnolo per lo Studio dei Tumori (IRST) “Dino Amadori,” Meldola, Italy; 8 Department of Medical and Surgical Sciences, University of Bologna, Bologna, Italy; 9 IRCCS Istituto Tumori “Giovanni Paolo II,” Bari, Italy; 10 Carlo Poma Hospital, ASST Mantova, Mantova, Italy; 11 Medical Oncology Unit, Fondazione IRCCS Ca’ Granda Ospedale Maggiore Policlinico, Milano, Italy; 12 Medical Oncology, S. Anna Hospital, Torino, Italy; 13 Breast Unit Ausl Modena, Ramazzini Hospital, Carpi, Italy; 14 IRCCS Istituto Romagnolo per lo Studio dei Tumori (IRST) “Dino Amadori,” Meldola, Italy; 15 Rete Clinica Senologica-Oncologia Medica S. Chiara, APSS, Trento, Italy; 16 Department of Medical and Surgical Sciences for Children and Adults, University Hospital of Modena, Modena, Italy; 17 Azienda Ospedaliero-Universitaria di Modena, Modena, Italy; 18 Pathology Unit, Ulss 5 Polesana, Rovigo, Italy; 19 Pathology, Microbiology and Immunology, Vanderbilt University Medical Center, Nashville, Tennessee; 20 University of Modena and Reggio Emilia, Modena, Italy

## Abstract

**Importance:**

For patients with early *ERBB2 *(formerly *HER2*)–positive breast cancer, there is a need to identify biomarkers to guide treatment de-escalation.

**Objective:**

To evaluate the association of tumor-infiltrating lymphocytes (TILs) with distant disease-free (DDFS) and overall survival (OS) for patients with *ERBB2*-positive early breast cancer.

**Design, Setting, and Participants:**

The ShortHER randomized clinical trial was a multicentric trial in Italy that enrolled patients with *ERBB2*-positive breast cancer from December 2007 to October 2013. Patients received 9 weeks or 1 year of adjuvant trastuzumab combined with chemotherapy. Tumor samples were evaluated for TILs. Herein, patients were evaluated at a median follow-up of 9 years, and data were analyzed from February 2023 to August 2024.

**Intervention:**

Four cycles of anthracycline-based chemotherapy followed by 4 courses of taxanes combined with trastuzumab for 1 year (long arm) or 3 courses of taxanes combined with trastuzumab for 9 weeks followed by reduced-dose anthracycline-based chemotherapy for 3 courses (short arm).

**Main Outcomes and Measures:**

The association of TILs with DDFS and OS was assessed with Cox models.

**Results:**

Of 1253 patients enrolled in the ShortHER trial, 866 women (median [IQR] age, 56 [48-64] years) had evaluable TILs. In Cox models with relevant factors, each 5% TIL increment was associated with improved DDFS (hazard ratio [HR], 0.87; 95% CI, 0.80-0.95; *P* = .001) and OS (HR, 0.89; 95% CI, 0.81-0.98; *P* = .01). The 10-year OS rate was 91.3% for patients with TILs 20% or higher, 93.3% for patients with TILs 30% or higher, and 98.1% for patients with TILs 50% or higher, resulting higher vs lower TIL counterparts. Patients with TILs lower than 20% showed a better outcome with the long vs short treatment (10-year DDFS, 88.7% vs 81.0%), whereas patients with TILs 20% or higher showed the opposite (10-year DDFS, 87.1% vs 92.2%; *P *for interaction = .01). Similarly, patients with TILs 20% or higher had a 10-year OS rate of 89.3% in the long arm vs 93.1% in the short arm (HR, 0.36; 95% CI, 0.10-1.36); patients with TILs lower than 20% had a 10-year OS rate of 91.3% in the long arm vs 86.9% in the short arm (HR, 1.36; 95% CI, 0.82-2.23; *P *for interaction = .06).

**Conclusions and Relevance:**

This follow-up analysis of the ShortHER randomized clinical trial is, to our knowledge, the first demonstration of an independent effect of TILs in terms of OS for patients with *ERBB2*-positive early breast cancer treated with adjuvant chemotherapy and anti-*ERBB2* therapy. Patients with TILs 20% or higher who de-escalated trastuzumab duration and chemotherapy dose were not exposed to an excess risk of distant relapse or death.

**Trial Registration:**

EudraCT: 2007-004326-25

## Key Points

**Question:**

Is the level of tumor-infiltrating lymphocytes (TILs) associated with survival outcomes in patients with *ERBB2 *(formerly *HER2*)–positive early breast cancer, and can it guide adjuvant treatment de-escalation?

**Findings:**

In this 10-year follow-up analysis of the ShortHER randomized clinical trial, among 866 patients with available TILs there was a statistically significant association in each 5% increase in TILs and improved overall survival. Patients with TILs 20% or higher were not exposed to excess risk of death when treated with de-escalated chemotherapy and trastuzumab.

**Meaning:**

TILs can serve as a biomarker to identify patients with *ERBB2*-positive early breast cancer who may safely undergo de-escalated adjuvant therapy without compromising long-term survival outcomes.

## Teaser

This 10-year follow-up analysis of the ShortHER randomized clinical trial evaluates the association of tumor-infiltrating lymphocytes with distant disease-free and overall survival among patients with *ERBB2* (formerly *HER2*)–positive early breast cancer.

## Introduction

Outcomes for patients with early *ERBB2 *(formerly *HER2*)–positive breast cancer have improved in recent decades because of new effective treatments. The current abundance of drugs and strategies that play a role in this disease, along with the consideration that most of the patients reach optimal survival outcomes at long term, mandates for careful treatment personalization based on the individual risk–benefit ratio. In such scenarios, there is an ongoing need to identify biomarkers that could help refine prognostic stratification and guide treatment de-escalation.

The ShortHER trial was a phase 3 noninferiority randomized clinical trial comparing chemotherapy combined with 1 year (long arm) vs 9 weeks (short arm) of adjuvant trastuzumab in patients with *ERBB2*-positive early breast cancer.^1^ While the primary disease-free survival (DFS) analysis did not allow for the noninferiority of the short arm, the recently published 10-year overall survival (OS) update shows quite superimposable outcomes for the 2 arms, especially for patients with no or limited nodal involvement.^2^

Previously, we reported on the possible prognostic role of tumor-infiltrating lymphocytes (TILs) in the ShortHER trial at a median follow-up of 6 years, showing a positive, independent association between TIL density and distant disease-free survival (DDFS) (10% increments: hazard ratio [HR], 0.73; 95% CI, 0.59-0.89).^3^ Notably, we observed a statistically significant interaction between TIL levels at a 20% or higher cutoff and treatment arm, as patients with low TILs showed a statistically significant benefit from the longer treatment (short vs long: HR, 1.75; 95% CI, 1.09-2.80), whereas those with high TILs had an excellent prognosis regardless of treatment duration, with even numerically better outcome in the short arm (short vs long: HR, 0.23; 95% CI, 0.05-1.09).

Our previous analysis added to a growing body of evidence supporting the prognostic value of TILs in *ERBB2*-positive breast cancer in both the neoadjuvant^4,5,6,7,8,9^and adjuvant setting,^10,11,12^ where TILs are strongly associated with surrogate end points such as increased rates of pathological complete responses and reduced risk of relapse. However, to our knowledge, no single study has demonstrated an association between TILs and OS, the most important survival end point in the early setting. Herein, we present the updated analysis of the possible prognostic role of TILs in the ShortHER trial and assess the impact of this biomarker on survival outcomes, including OS, at a long-term follow-up.

## Methods

### Study Design, Patients, and Treatments

The ShortHER trial (EudraCT: 2007-004326-25; ClinicalTrials.gov Identifier: NCT00629278) was a multicenter, phase 3 noninferiority study conducted in Italy. Details of the trial design, statistical power, and eligibility criteria have been previously published (see also Supplement 1).^1^

The trial randomized 1254 patients with *ERBB2*-positive early breast cancer to receive 9 weeks (short arm; experimental) vs 1 year (long arm) of adjuvant trastuzumab combined with chemotherapy. Notably, the chemotherapy regimen differed between treatment arms. In the long arm, patients received doxorubicin, 60 mg/m^2^, plus cyclophosphamide, 600 mg/m^2^, or epidoxorubicin, 90 mg/m^2^, plus cyclophosphamide, 600 mg/m^2^, administered once every 3 weeks for 4 courses followed by paclitaxel, 175 mg/m^2^, or docetaxel, 100 mg/m^2^, once every 3 weeks for 4 courses; in the short arm, chemotherapy consisted of docetaxel, 100 mg/m^2^, once every 3 weeks for 3 courses followed by fluorouracil, 600 mg/m^2^, epidoxorubicin 60, mg/m^2^, and cyclophosphamide, 600 mg/m^2^, administered once every 3 weeks for 3 courses.

The primary end point of the trial was DFS. OS was analyzed as a second primary outcome.

At the primary DFS analysis (median follow-up of 6 years), the study did not demonstrate the noninferiority of the short arm, showing a 5-year DFS of 88% in the long arm and 85% in the short arm, with the upper border of the CI for the HR of 1.13 (90% CI, 0.89-1.42) crossing the upper limit of 1.29, which was chosen as the noninferiority margin.^1^ At the more recent median follow-up of 9 years, the 10-year DFS was 77% in the long arm and 78% in the short arm (HR, 1.06; 90% CI, 0.86-1.31); the 10-year OS was 89% in the long arm and 88% in the short arm (HR, 1.15; 90% CI, 0.85-1.56).^2^

This trial was approved by local ethical committees and conducted in compliance with the principles of Good Clinical Practice and the Declaration of Helsinki. Patients provided written informed consent for tumor sample use for research purposes. The trial followed the Consolidated Standards of Reporting Trials (CONSORT) reporting guidelines.

### Pathology

TILs were centrally assessed on a single hematoxylin-eosin–stained slide of the primary tumor tissue according to guideline recommendations.^7^ Details on the TIL assessment, including concordance between the 2 investigators (M.V.D. and M.S.) involved in scoring, have been previously reported.^3^

*ERBB2* and hormone receptor status were assessed locally. Hormone receptor–negative status was defined as estrogen receptor and progesterone receptor expression less than 10%.

### Statistical Analysis

The population for the TIL analysis is based on the total number of patients with centralized tumor samples suitable for the analysis (866 per protocol). Therefore, no formal sample-size calculation was carried out.

DDFS was calculated from the time from randomization until distant relapse or death, whichever occurred first.^13^ OS was calculated from the time of randomization until the last follow-up or death, whichever occurred first. Patients without events were censored at the time of the last follow-up.

For survival analysis, TILs were primarily considered as a semicontinuous variable (5% increments), and the Cox regression model was used to calculate HRs and 95% CIs. The Kaplan-Meier method was used to estimate survival curves, and the log-rank test was used to compare survival curves, adopting different arbitrary cutoffs to define patients with low or high TILs (≥20%, ≥30%, and ≥50%). The interaction between TILs and treatment was explored at the 20% or higher cutoff to define high- vs low-TIL groups, to maintain consistency with the previously published analysis.^3^ At that time, this cutoff was arbitrarily chosen to identify a group of patients with a DDFS rate at 5 years of at least 95%, which was considered a clinically acceptable definition of low-risk patients. To study the interaction between treatment arm and TILs, we used a Cox model, including treatment arm, TILs, and the interaction term.

Data were analyzed from February 2023 to August 2024. Statistical analyses were performed using SPSS, version 29 (IBM). All tests were 2-sided, with *P* < .05 indicating statistical significance.

## Results

### Patients’ Characteristics and Follow-Up

**Figure 1.**
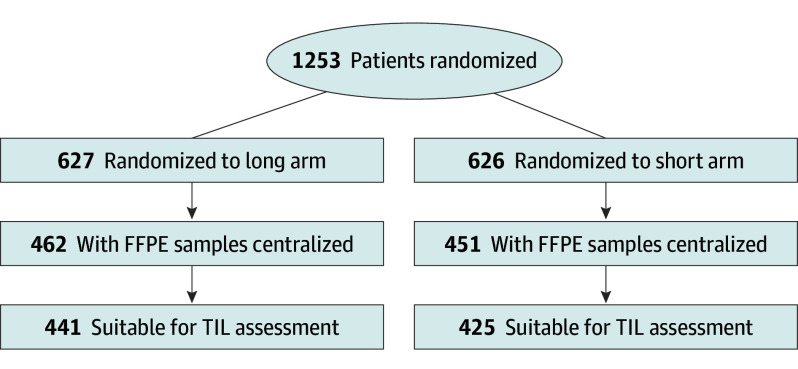
CONSORT Flowchart for the Tumor-Infiltrating Lymphocytes (TILs) Substudy of the ShortHer Trial FFPE indicates formalin-fixed paraffin embedded.

Patients characteristics have been previously published.^3^ Briefly, 866 women (median [IQR] age, 56 [48-64] years) with available TILs were included in the analysis (TIL cohort), constituting 69% of all patients randomized in the ShortHER trial (Figure 1). Clinical-pathological features of the cohort with TILs were representative of the broader study cohort and similar to patients without available TILs, except for a slightly younger age in the cohort with TILs.^3^ Median (IQR) TIL density was 5% (1%-15%).

At a median follow-up of 9.02 years (95% CI, 9.00-9.04 years), the total number of events was 107 for DDFS (24 more compared to the previously published analysis^3^) and 74 events for OS. The HR for the DDFS comparison between the short vs long arms was 1.26 (95% CI, 0.92-1.72) in all randomized patients and 1.44 (95% CI, 0.98-2.10) in the cohort with TILs. The HR for the OS comparison between the short vs long arms was 1.15 (95% CI, 0.80-1.65) in all randomized patients.^2^ The HR for OS in the cohort with TILs was similar (1.11; 95% CI, 0.71-1.76).

### TILs and Prognosis

The level of TILs was statistically significant and independently associated with both DDFS and OS. In Cox models including relevant variables (age, menopausal status, anatomic stage, histologic grade, and hormone receptor status), for every 5% increase in TILs there was a 13% reduction in the risk of a DDFS event (HR, 0.87; 95% CI, 0.80-0.95; *P* = .001) and a 11% reduction in the risk of death (HR, 0.89; 95% CI, 0.81-0.98, *P* = .01). The other variable that maintained an independent association was pathologic stage (Table).

**Table.** Cox Regression Models for Distant Disease-Free and Overall Survival

Variable	Distant disease-free survival		Overall survival	
	Hazard ratio (95% CI)	*P* value	Hazard ratio (95% CI)	*P* value
Tumor-infiltrating lymphocytes, 5% increase	0.87 (0.80-0.95)	.001	0.89 (0.81-0.98)	.01
Age (continuous)	1.02 (0.99-1.05)	.12	1.03 (0.99-1.06)	.11
Menopausal status (postmenopausal vs premenopausal)	0.71 (0.37-1.33)	.28	1.13 (0.52-2.47)	.76
Grade 3 vs 1-2	1.36 (0.87-2.15)	.18	1.60 (0.90-2.81)	.11
Stage II vs I	1.36 (0.87-2.15)	.18	1.10 (0.63-1.91)	.75
Stage III vs I	3.60 (2.17-5.92)	<.001	2.86 (1.61-5.08)	<.001
Hormone receptor status (positive vs negative)	0.75 (0.49-1.14)	.18	0.82 (0.50-1.36)	.45

**Figure 2.**
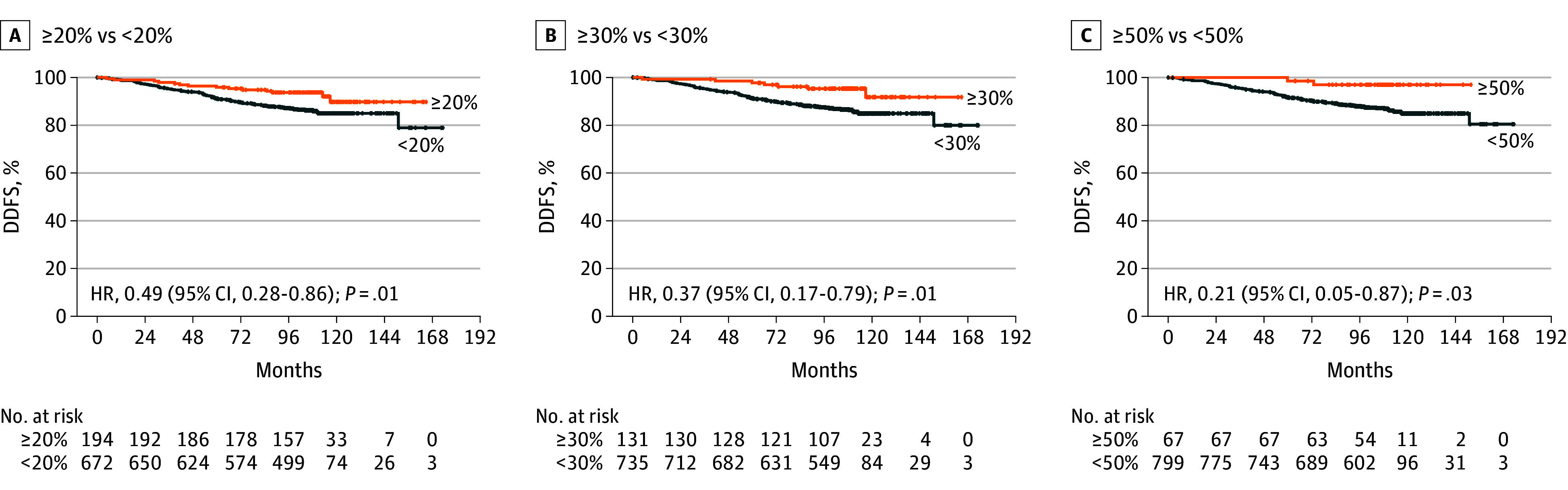
Kaplan-Meier Curves for Distant Disease-Free Survival (DDFS) in Patients With High vs Low Tumor-Infiltrating Lymphocytes High values are those at the cutoff or higher, and low values are those below the cutoff. HR indicates hazard ratio.

**Figure 3.**
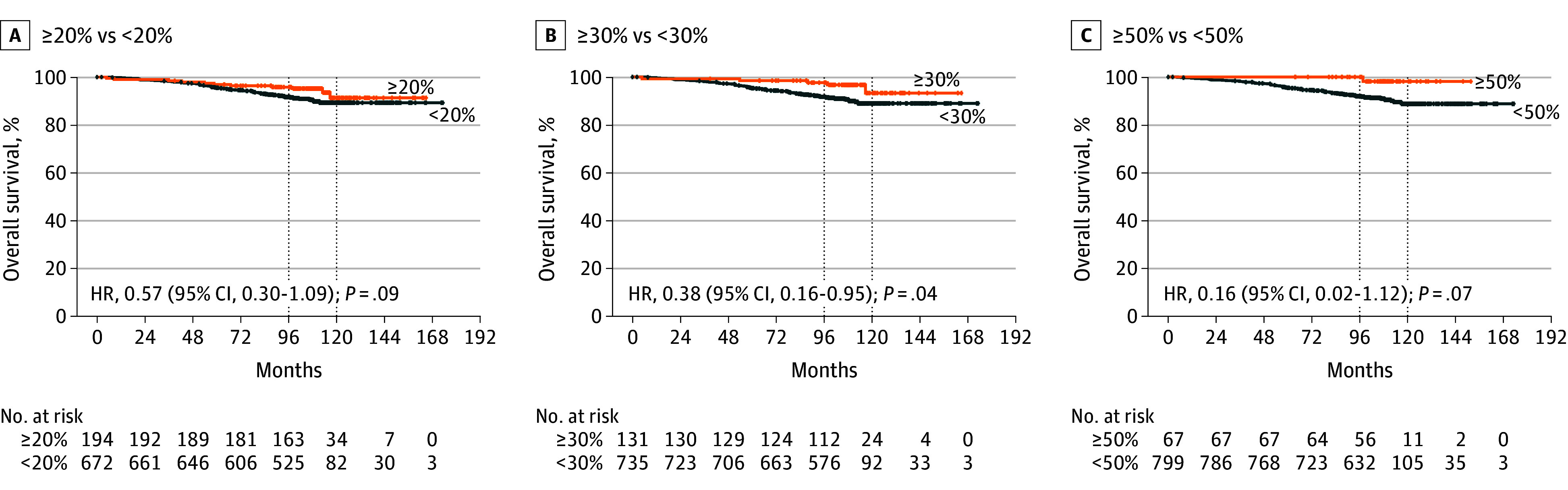
Kaplan-Meier Curves for Overall Survival in Patients With High vs Low Tumor-Infiltrating Lymphocytes High values are those at the cutoff or higher, and low values are those below the cutoff. Dotted lines indicate the 8- and 10-year survival time points. HR indicates hazard ratio.

Next, patients were stratified according to TIL levels, and survival curves were compared for patients with high and low TILs, according to different cutoffs (<20% vs ≥20%, <30% vs ≥30%, and <50% vs ≥50%). Figures 2 and 3 report Kaplan-Meier curves for DDFS and OS according to these cutoffs. In terms of DDFS, patients with high TILs experienced a statistically significant better outcome compared to patients with lower TILs at each considered cutoff. The absolute difference in both 8-year and 10-year DDFS rates was cutoff dependent, reaching 8.9% and 12.0% in DDFS and OS, respectively, at the 50% cutoff point. The 10-year DDFS rates for patients in the high-TIL groups reached 89.8% for patients with TILs 20% or higher, 91.7% for patients with TILs 30% or higher, and 96.9% for patients with TILs 50% or higher. Similarly, there was a numerically better OS for patients with high vs low TILs at each considered cutoff point, reaching statistical significance for patients with TILs 30% or higher vs lower (HR, 0.38; 95% CI, 0.16-0.95, *P* = .04). Similarly to what was observed for DDFS, the absolute difference in both 8-year and 10-year OS rates progressively increased at higher cutoff points, reaching 6.1% and 9.3% in DDFS and OS, respectively, at the 50% cutoff point. The 10-year OS rates for patients in the high-TIL groups were cutoff dependent, reaching 91.3% for patients with TILs 20% or higher, 93.3% for patients with TILs 30% or higher, and 98.1% for patients with TILs 50% or higher.

### Interaction Between TILs and Treatment Arm

There was a statistically significant association between TILs as a semicontinuous variable (5% increase) and DDFS and OS in patients from the short arm (DDFS: HR, 0.82; 95% CI, 0.72-0.94; *P* = .003; OS: HR, 0.86; 95% CI, 0.75-0.99; *P* = .04), though not in patients from the long arm (DDFS: HR, 0.93; 95% CI, 0.84-1.03; OS: HR, 0.94; 95% CI, 0.84-1.05), with nonstatistically significant interaction tests (DFFS: *P* for interaction  = .122; OS: *P* for interaction = .39). At this updated analysis, the observation of a statistically significant interaction between treatment arm and TILs was confirmed at the 20% cutoff for DDFS (*P *for interaction = .01), with patients with low TILs showing a statistically significant better outcome with the long vs short treatment (10-year DDFS, 88.7% vs 81.0%; *P* = .006), while patients with high TILs showed an excellent outcome irrespective of treatment arm, with even numerically improved DDFS when treated in the short vs long arm (10-year DDFS, 92.2% vs 87.1%; *P* = .09).

**Figure 4.**
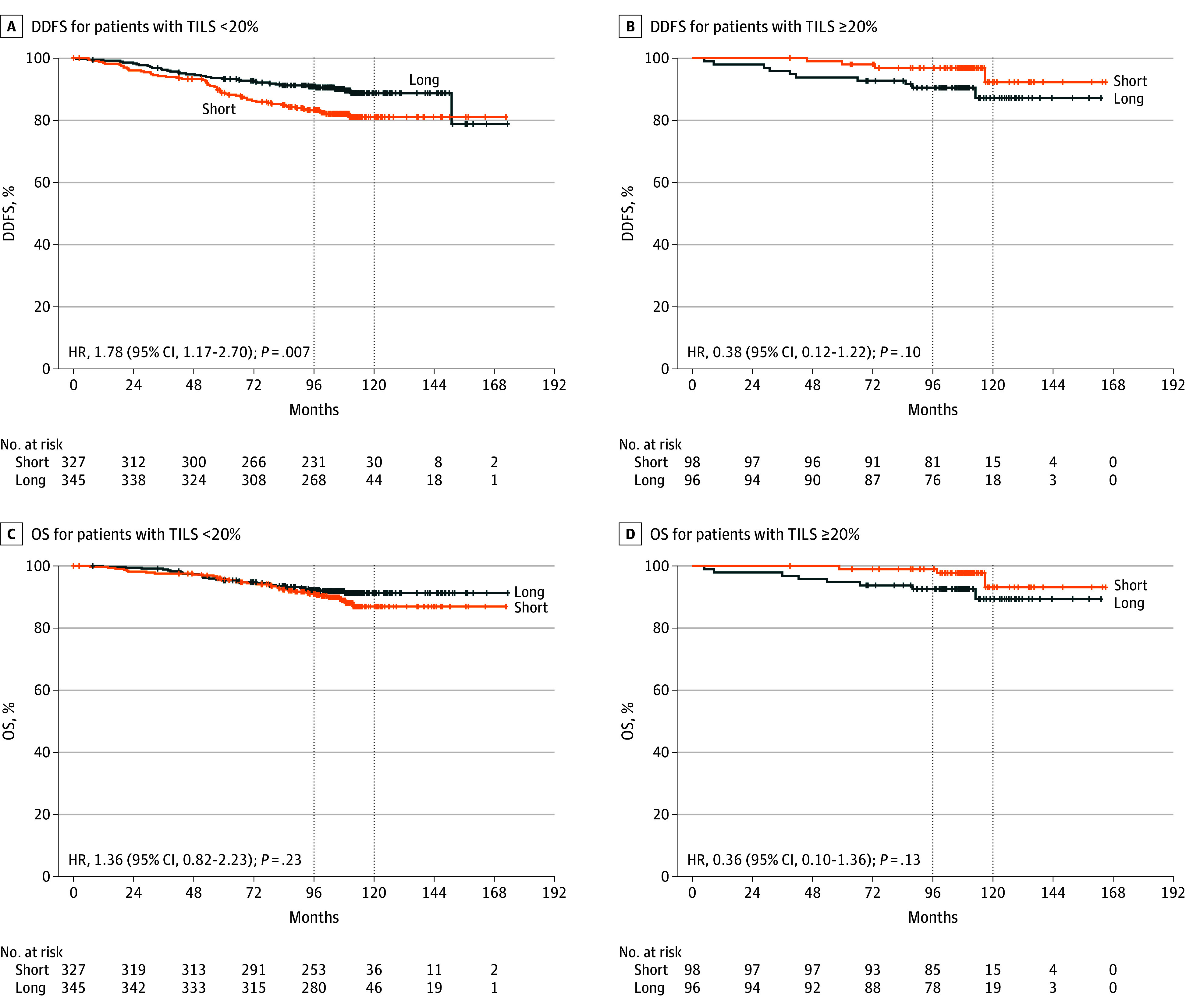
Interaction Between Tumor-Infiltrating Lymphocytes (TILs) and Treatment Arm Kaplan-Meier curves for distant disease-free survival (DDFS) compare the 2 treatment arms for patients with TILs lower than 20% (A) and patients with TILs 20% or higher (B) (*P* for interaction = .01). Kaplan-Meier curves for overall survival (OS) compare the 2 treatment arms for patients with TILs lower than 20% (C) and patients with TILs 20% or higher (D) (*P* for interaction = .06). Dotted lines indicate the 8- and 10-year survival time points. HR indicates hazard ratio.

Kaplan-Meier curves are shown in Figure 4A and B. The results of the interaction analysis considering OS as the clinical outcome pointed toward the same direction (Figure 4C and D). For patients with TILs lower than 20%, there was a numerical advantage for the long vs short treatment (10-year OS, 91.3% vs 86.9%; HR, 1.36; 95% CI, 0.82-2.23; *P* = .23). Patients with TILs 20% or higher showed a numerically better OS in the short vs long arm (10-year rate, 93.1% vs 89.3%; HR, 0.36; 95% CI, 0.10-1.36; *P* = .13), with *P* for interaction of .06).

### Interaction Between TILs and Stage

To further explore the independent roles of TILs and stage, as these were the only 2 factors emerging as having a statistically significant association with DDFS and OS in Cox models (Table), the association between TILs and survival was evaluated in stage-defined subgroups. Results showed a statistically significant impact of TILs only for patients with stage II disease, for both DDFS and OS. The HR for DDFS for each 5% increment in TILs was 0.98 (95% CI, 0.88-1.09) for stage I, 0.73 (95% CI, 0.59-0.91; *P* = .004) for stage II, and 0.89 (95% CI, 0.78-1.03; *P* = .11) for stage III (*P* for interaction = .23). For OS, the HR for each 5% increment in TILs was 1.01 (95% CI, 0.91-1.12) for stage I, 0.69 (95% CI, 0.51-0.95; *P* = .02) for stage II, and 0.88 (95% CI, 0.74-1.05) for stage III (*P *for interaction = .07). Kaplan-Meier OS curves for patients with TILs 20% or higher vs TILs lower than 20% in different stage groups are shown in the eFigure in Supplement 2.

## Discussion

This updated analysis from the ShortHER trial provides, to our knowledge, the first evidence of an independent effect of TILs on OS in patients with *ERBB2*-positive early breast cancer treated with adjuvant chemotherapy and trastuzumab. We demonstrated at a median follow-up of 9 years that there was a statistically significant association between increased TIL density and improved OS and DDFS. Moreover, these data suggest that patients with high TILs at the 20% or higher cutoff who de-escalate trastuzumab duration and chemotherapy dose are, at least, not exposed to an excess risk of death, indicating that TILs might be used to identify a subgroup of patients who can safely receive less intensive treatment.

The prognostic role of TILs in early *ERBB2*-positive breast cancer is supported by growing evidence.^14,15^ In the neoadjuvant setting, high levels of TILs are associated with increased rates of pathological complete responses and reduced risk of relapse.^4,5,6,7,8,9^ In the adjuvant setting, TIL density is associated with improved outcomes, though previous studies have focused on surrogate end points rather than OS.^10,11,12^ Recently, a large individual data meta-analysis by the Early Breast Cancer Trialists’ Collaborative Group, including 4168 patients from 5 randomized pivotal trials of adjuvant trastuzumab with chemotherapy vs chemotherapy alone, confirmed the important prognostic role of TILs in terms of time to first recurrence, but no OS data were reported.^16^ The present study extends these findings by demonstrating a direct independent association between TILs and the most valuable survival end point, strongly reinforcing the role of TILs in patients with early *ERBB2*-positive breast cancer.

The question about the optimal TIL cutoff remains open, since in this study (and others^16^) there is no evidence of a natural cutoff that maximizes the possible prognostic potential of TILs. TILs are associated with survival as a continuous variable; therefore, the higher the cutoff point, the higher the ability of the high-TIL definition to identify patients with an excellent outcome. Moreover, in this study, the slope of the curves for low vs high TIL levels are different, especially when using higher cutoffs, suggesting that the time course of the disease is fundamentally altered rather than just delayed. Although the absence of a clear prognostic cutoff might put into discussion the translation of TILs into clinical practice, 2 arguments can be raised. First, it is important to define the cutoff that best supports clinicians in a specific treatment choice, which is not necessarily the best prognostic cutoff. This question is optimally addressed in trials testing de-escalation approaches, as in the ShortHER trial. Second, TILs could be considered as a continuous variable in integrated prognostic tools.

Regarding the first point, we have demonstrated a statistically significant interaction between TILs at the 20% or higher cutoff and treatment arm, as patients with low TILs showed a better outcome with the standard long treatment, while patients with high TILs not only had an excellent prognosis regardless of treatment duration, but also the outcome was numerically improved in case of treatment in the short arm. This observation suggests that TILs can help identify patients who may be optimally treated with de-escalated treatment. Although the benefit of standard de-escalation overtreatment in patients with low TILs, and therefore at worse prognosis, is more easily explainable, the reasons for observing an apparent improved survival for patients with high TILs receiving the shorter therapy are less straightforward. One could speculate that, in presence of high TILs at baseline, a full treatment, including higher doses of immunogenic drugs (anthracyclines and trastuzumab), might run the risk of exhausting the immune response, with potential detrimental effects at long term. However, this remains a hypothesis to be verified.

Previous adjuvant studies have produced mixed results regarding the predictive role of TILs for trastuzumab benefit.^10,11^ The recent Early Breast Cancer Trialists’ Collaborative Group meta-analysis did not find any interaction between TILs and trastuzumab treatment, meaning that the benefit of adding adjuvant trastuzumab to chemotherapy was independent from the level of TILs.^16^ The present data require external validation; therefore, we cannot conclude that high TILs are predictive of a better efficacy of treatment de-escalation. However, with a positive interaction test and given the demonstrated effect of TILs, it is highly unlikely that the outcome of patients in the high-level TIL group receiving de-escalation would have, in the end, worse outcomes than those of patients with high TILs receiving standard treatment. For this reason, we conclude that patients with TILs 20% or higher receiving a reduced anthracycline dose and trastuzumab duration in this study were at least not exposed to an excess risk of distant relapse or death. Fitting with the topic of anthracycline de-escalation/omission, the TRAIN-2 trial showed no difference in pathological complete response rate and invasive DFS between neoadjuvant treatment with anthracyclines and taxane-containing chemotherapy vs anthracycline-free chemotherapy, combined with trastuzumab and pertuzumab.^5^ Although there was no interaction between TILs and treatment arm in terms of invasive DFS, it is worth mentioning that patients with TILs lower than 14% (which is close to the ≥20% cutoff used for interaction in this study) showed a numerically better outcome if treated with the anthracycline-free regimen compared to the anthracycline-containing regimen (invasive DFS rate at 3 years, 96.0% vs 92.6%). These data, although based on a low number of patients and derived from a trial dedicated to a different setting and addressing a different clinical question than ShortHER, show points of convergence with our work.

The best approach for prognostic patient stratification involves the integration of multiple parameters. Currently, the clinical management of patients with *ERBB2*-positive early breast cancer relies on clinical-pathological staging,^17^ with limited use of biomarkers for risk stratification besides *ERBB2 *status.^18^ De-escalation of adjuvant therapy is already a reality for most patients with stage I *ERBB2*-positive breast cancer. In this study, the effect of TILs was predominantly observed for patients with stage II disease, for whom the implications of treatment de-escalation are more debated. The present findings advocate for the inclusion of TILs in integrated prognostic tools in this setting, providing a reproducible,^19^ cost-effective, and readily worldwide available measure contributing to the identification of patients with an excellent prognosis who may derive no harm from de-escalation of adjuvant treatment.

### Strengths and Limitations

Strengths and limitations of this study have been acknowledged previously.^3^ The strength of the present update lies in the long-term follow-up. Limitations include the exploratory nature of the analysis, which was not prespecified in the protocol.

## Conclusions

This updated analysis of the ShortHER randomized clinical trial is, to our knowledge, the first report of a strong independent association of TILs with OS for patients with *ERBB2*-positive early breast cancer. Validation is needed to confirm that patients with high TILs can safely undergo treatment de-escalation. These findings support the integration of immune-related features into prognostic tools for *ERBB2*-positive early breast cancer, moving toward personalized treatment strategies beyond clinical-pathological features.

## Notes

Supplement 1. Trial Protocol

Supplement 2. **eFigure.** Kaplan-Meier overall survival curves comparing patients with high TILs (>20%) vs patients with lower TILs (<20%) in stage-defined groups

Supplement 3. Data Sharing Statement
