# Supplementary material for: Tumor-Infiltrating Lymphocytes and Survival Outcomes in Early ERBB2-Positive Breast Cancer: 10-Year Analysis of the ShortHER Randomized Clinical Trial
Source: JAMA Oncol. 2025 Feb 13;11(4):386–93. doi: 10.1001/jamaoncol.2024.6872 (PMC11826437; doi:10.1001/jamaoncol.2024.6872)
Supplement: Supplement 1. — Trial Protocol [file jamaoncol-e246872-s001.pdf]

SHORT-HER: MULTICENTRIC RANDOMIZED PHASE III TRIAL OF 2 DIFFERENT ADJUVANT CHEMOTHERAPY REGIMENS PLUS 3 VS 12 MONTHS OF TRASTUZUMAB IN HER2 POSITIVE BREAST CANCER PATIENTS

EudraCT Number 2007-004326-25

NCI: NCT00629278

Version n. 7 - 20 Jun. 2015

Sponsor:
Dept. of Surgery, Oncology and Gastroenterology – University of Padova, Italy

Financial Supporter:
Agenzia Italiana del Farmaco (AIFA) - Roma

Coordinating Center:
Medical Oncology 2
Dept. of Surgery, Oncology and Gastroenterology – University of Padova, Italy

Principal Investigator
Prof. PierFranco Conte
Medical Oncology 2
Dept. of Surgery, Oncology and Gastroenterology – University of Padova, Italy

Steering Committee
- PIERFRANCO CONTE (CHAIRMAN)
Dept. of Surgery, Oncology and Gastroenterology – University of Padova, Italy

- ANTONIO FRASSOLDATI (CCR Emilia Romagna)
AOU Sant'Anna Ferrara

- VALENTINA GUARNERI
Dept. of Surgery, Oncology and Gastroenterology – University of Padova, Italy

- ANNAMARIA MOLINO (CCR Veneto, Trentino, Friuli)
Università di Verona, Ospedale Maggiore - Dept Of Oncology

- ROBERTO D'AMICO (Statistical Committee)
Dept. of Oncology nnd Hematology- University of Modena and Reggio Emilia, Modena

GIANCARLO BISAGNI
Az Ospedaliera Reggio nell'Emilia – Reggio Emilia

- ALBA BRANDES
Az Ospedalieria Bellaria – Bologna

- LUIGI CAVANNA
Az Ospedaliera Piacenza - Piacenza

- FRANCESCO GIOTTA
Istituto Tumori Giovanni Paolo II – Bari

-MICHELA DONADIO
Az Ospedaliera Universitaria San Giovanni Battista – Torino

- ORNELLA GARRONE
ASO. S. Croce E Carle – Cuneo

-ANTONIO RUSSO
Policlinico Giaccone – Palermo

-GIAN LUCA DE SALVO
IRCCS Istituto Oncologico Veneto - Padova

Independent Data Monitoring Committee
Edith Perez,
Div. of Hematology/Oncology, Mayo Clinic, Jacksonville, FL

Michel Marty,
Hospital Saint Louise, Paris, FR

Mahesh Parmar,
Medical Research Council, London, UK

Statistical Committee
Roberto D'Amico
Dept. of Oncology and Hematology- University of Modena and Reggio Emilia, Modena

Paola Del Bianco
IRCCS Istituto Oncologico Veneto – Padova

Oriana Nanni
Romagna Cancer Institute

Paolo Bruzzi
Clinical Epidemiology and Trials, National Cancer Research Institute, Genova

Trial Office
Roberto Vicini (Information technology)
Dept. of Oncology and Hematology- University of Modena and Reggio Emilia, Modena

Laura McMahon (Study Coordinator)
Istituto Oncologico Veneto IRCCS Padova, Italy

Scientific Secretary

Valentina Guarneri
University of Padova, Italy

Laura McMahon
Istituto Oncologico Veneto IRCCS Padova, Italy

TABLE OF CONTENTS

PROTOCOL SYNOPSIS ______ 6

LIST OF ABBREVIATIONS AND DEFINITION OF TERMS ______ 10

1 BACKGROUND AND RATIONALE ______ 11

2. STUDY OBJECTIVES ______ 14

2.1 Primary objective 14

2.2 Secondary objectives 14

3. STUDY PLAN AND PROCEDURES ______ 14

3.1 Overall study design and flow chart 14

3.2 Study Procedures 15

3.3 Laboratory assessment 16

3.4 Pregnancy Test 17

3.5 Serum ECD determination 17

3.6 Selection of study population 20

3.6.1 Study selection record 20
3.6.2 Inclusion criteria 20
3.6.3 Exclusion criteria 20
3.6.4 Restrictions 21

3.7 Discontinuation of subjects from treatment and assessment 21

3.7.1 Criteria for discontinuation 21
3.7.2 Procedures for discontinuation 21

3.8 Treatments 21

3.8.1 Investigational products 21
3.8.2 Doses and treatment regimens 22
3.8.3 Treatment details 22
3.8.4 Prophylactic use of Colony stimulating factors (G-CSF) 22
3.8.5 Storage 22
3.8.6 Method of assigning subjects to treatment group 22

3.9 Toxicity 23

3.9.1 Cardiac toxicity 23
3.9.2 Chemotherapy dose reduction/interruption 24
3.9.3 Trastuzumab dose interruption/delayed 25

3.10 Study Closure 25

4. MEASUREMENTS OF STUDY VARIABLES AND DEFINITIONS OF OUTCOME VARIABLES ______ 26

4.1 Disease free survival 26

4.2 Overall survival 26

4.3 Failure Rate ... 26
4.4 Incidence of cardiac events ... 26
4.5 Safety Profile ... 26
4.5.1 Adverse Events (AE) and serious Adverse events (SAE) 26
4.6 Time Period, Frequency, and Method of Detecting AEs and SAEs ... 29
4.6.1 Recording of AEs and SAEs 29
4.6.2 Evaluating AEs and SAEs 30
4.6.2.1 Assessment of Intensity 30
4.6.2.2. Assessment of Causality 30
4.6.2.3 Follow-Up of AEs and SAEs 30
4.7 Reporting of SAEs to National Coordinating Centre (NCC) ... 31
4.7.1 Timeframes for Submitting SAE Reports to NCC 31
4.7.2 Regulatory Reporting Requirements For Adverse Events 31
5. DATA MANAGEMENT 32
6. STATISTICAL METHODS AND DETERMINATION OF SAMPLE SIZE 32
7. STUDY MANAGEMENT 34
7.1 Monitoring ... 34
7.2 Changes to the protocol ... 35
8. ETHICS 35
8.1 Ethics review ... 35
8.2 Ethical conduct of the study ... 35
8.3 Written informed consent ... 35
8.4 Subject data protection ... 35
9. REFERENCES 36

PROTOCOL SYNOPSIS

Study phase
Randomized phase III trial.

Study population
Surgically resected HER2-neu positive women with early breast cancer

Study centers
The trial is a multicenter study conducted at several Italian Oncology Services.
The coordination of the study is performed by the Medical Oncology 2 Dept. of Surgery, Oncology and Gastroenterology – University of Padova, Italy

Study period
Estimated date of first subject enrolled: October 2007
Estimated date of last subject enrolled: November 2012

Objectives
Primary
- Primary objective is to evaluate if 3 months (9 weekly administrations) of Herceptin (H) administered according to the Finnish protocol are not inferior to 12 months (18 three-weekly administrations) in a standard chemotherapy protocol in term of disease free survival, in patients with HER2 positive early breast cancer.
- Second primary end point is overall survival. The study is designed assuming that prolonged H administration concomitantly with chemotherapy produces more frequent cardiac events than shorter H treatment, and that the onset of this potentially serious toxicity might preclude the benefit of treatment to a significant proportion of patients.

Secondary
-Failure Rate (FR) at 2 yrs (relapse, contralateral breast tumor (excluding DCIS) , death for any cause, treatment discontinuation due to toxicity)
-Cardiac events (absolute decrease of LVEF > 15%, absolute decrease of LVEF > 10% below 50%, CHF, other cardiac toxicities)

Study design
Multicenter, randomized phase III trial conducted in HER2-neu positive surgically resected breast cancer patients.
Eligible patients will be randomized to:
- Treatment Arm A (Long): Chemotherapy plus Trastuzumab (concomitant and sequential) for a total 18 3-wkly doses of H during 12 months
- Treatment Arm B (Short): Chemotherapy plus Trastuzumab weekly for the first 3 months (9 doses) .

Chemotherapy regimen
Chemotherapy regimens including an anthracycline and a taxane can be considered applicable as adjuvant therapy, on the basis of randomized clinical trials and consensus

statements. In particular, the regimen adopted as standard arm is the regimen used by NSAPB in the protocol B31 and by NCCTG in the protocol N9131, in which the efficacy of trastuzumab has been observed, with doxorubicin or epirubicin plus cyclophosphamide followed by paclitaxel or docetaxel.
Each center will define the regimen adopted before starting the trial enrollment.
The experimental arm is the regimen used in the FinHER trial, consisting in 3 courses of docetaxel followed by three courses of FEC (fluorouracil, epirubicin and cyclophosphamide)

Chemotherapy, dosage and mode of administration
Arm A
Investigators should administer either AC (adriamycin 60 mg/sqm plus cyclophosphamide 600 mg/sqm) or EC (epidoxorubicin 90 mg/sqm plus cyclophosphamide 600 mg/sqm) i.v infusion on day 1 every 21 days for 4 courses.
After 4 courses of AC or EC, patients will receive trastuzumab iv (8mg/kg loading dose starting with the 1st cycle of taxane, and 6 mg/kg thereafter) for 4 courses every 21 days and docetaxel or paclitaxel as follows:
- patients < 65 years: **docetaxel 100 mg/sqm** iv infusion over 1 hour with standard premedication **or paclitaxel 175 mg/sqm** iv infusion over 3 hours with standard premedication
- patients ≥ 65 years: **docetaxel 80 mg/sqm** iv infusion over 1 hour with standard premedication **or paclitaxel 175 mg/sqm** (**dose unchanged for paclitaxel**) iv infusion over 3 hours with standard premedication
At completion of the chemotherapy program, patients will receive trastuzumab monotherapy 6 mg/kg iv infusion every 21 days for 14 additional courses (for a total of 18 trastuzumab doses).

Arm B
- **Patients < 65 years** : **Docetaxel 100 mg/ sqm** iv on day 1 every 21 days for 3 courses plus trastuzumab (4 mg/kg loading dose at 1st administration only, followed by 2 mg/kg) weekly for 9 weeks starting together with the first dose of docetaxel.
- **Patients ≥ 65 years old**: **Docetaxel 80 mg/sqm** iv on day 1 every 21 days for 3 courses plus trastuzumab (4 mg/kg loading dose at 1st administration only, followed by 2 mg/kg) weekly for 9 weeks starting together with the first dose of docetaxel
At completion of docetaxel-trastuzumab, patients will receive 3 courses of FEC. The FEC regimen (5-Fluorouracil 600 mg/smq, Epidoxorubicin 60 mg/sqm, Cyclophospamide 600 mg/sqm, iv on day 1) will be started 21 days after the last docetaxel administration, and it will be delivered every 21 days for 3 courses.

Prophylactic use of Colony stimulating factors (G-CSF)
Patients ≥ 65 yrs receiving Docetaxel (80 mg/sqm) can receive prophylactic G-CSF from the first course at the discretion of treating physician.
Trastuzumab, dosage and mode of administration
In the control arm, trastuzumab will be administered concomitantly with taxane and sequential for a total 18 3-wkly doses of H during 12 months. The first dose will be 8 mg/kg, i.v. in 90 minutes, and will be administered on the day of the first taxane course. The subsequent administrations will be performed every 3 week, at the dose of 6 mg/kg, i.v., in 60 minutes
In the experimental arm, trastuzumab will be administered weekly for the first 3 months, concomitantly with docetaxel. The first dose will be 4 mg/kg, i.v. in 60 minutes, and will be

administered on the day of the first docetaxel course. The subsequent administrations will be performed weekly, at the dose of 2 mg/kg, i.v., in 30 minutes.

Duration of treatment

Treatment will last 14 months (4 3-wkly courses of AC or EC followed by 4 3-wkly taxane + H followed by 14 3-wkly H) in the control arm and 4 months (3 3-wkly docetaxel + 9 wkly H followed by 3 3-wkly FEC) in the experimental arm.
Study treatment will be stopped in case of disease progression, unacceptable toxicity, or withdrawal of consent.

Other Treatments

Radiotherapy will be given after chemotherapy and concomitantly with trastuzumab in the control arm to patients treated with conservative surgery, or with $\geq$ 4 positive axillary nodes. Patients with hormone-receptor positive tumor will receive hormonal therapy after chemotherapy, and concomitantly with trastuzumab in the control arm. Postmenopausal patients will be treated with an aromatase inhibitor for 5 years, premenopausal patients will be treated with an LH-RH agonist for 2 years plus tamoxifen for 5 years-.

Endpoint evaluation

° Disease free survival (DFS), calculated as the time interval between randomization and any of the following events, whichever first: local, regional and distant recurrence; contralateral breast cancer (excluding in situ carcinoma); other second primary cancer; death before recurrence or second primary cancer. Patients who will not experience relapse at the time of the last follow-up will be censored.
° Overall survival (OS) will be evaluated as second primary analysis outcome. The survival will be calculated as the time-interval between randomization and patient death or last follow-up.
Other Endpoints
° Failure Rate (FR) at 2 years, calculated as cumulative incidence of relapse, contralateral breast cancer (excluding in situ carcinoma), death for all causes, treatment withdrawal due to toxicity of therapy.
° Incidence of cardiac events (defined as decrease of EF > 15% from basal values, or decrease > 10% with EF absolute value below 50%, or symptomatic cardiac failure, or other cardiac side effects grade 2 or more according to NCI CTCAE (version 3, published
31 March 2003)

Centralized revaluation of Her2 status

Tumor tissue sample will be sent to the center of Modena for the centralized revaluation of Her2 status and then stored c/o the Pathology Institute of Policlinico of Modena. Any further use of the biological material cited above in new research projects regarding breast cancer will be planned and performed in the coordinating center and will be subject to approval of the Competent Ethical Committee. For this purpose the biological samples will be transferred from University of Modena to Oncologia Medica 2 - Istituto Oncologico Veneto, Dept. of Surgery, Oncology and Gastroenterology – University of Padova, Italy

Sample size

The analysis will take the form of a non inferiority test. The sample size calculation relates to the study's primary outcome which is the DFS. Let HR be the ratio between the hazard rate of events following short treatment and the hazard rate of events following long treatment. Formally, we define the short-treatment to be inferior to long-treatment if the hypothesis that HR ≥ 1.29 is true (null hypothesis), whereas, we define short to be non-inferior to long if HR [illegible]1.29 (alternative hypothesis). The sample size calculation has been amended in respect to the original protocol, due to the following reasons:

a) a slower than expected rate of enrolment has been observed, mainly due to delays attributable to the process necessary to activate centres;

b) less patients than expected were available for randomisation due to the presence of competitive studies in the centres involved in this project, mostly performed by the pharmaceutical industry;

Hence, it has been agreed to conclude the accrual of patients in November 2012. Taking into account the enrolment rate observed in the last months it is reasonable to state that the expect total number of patients enrolled by November 2012 will be 1250.

The study therefore will provide results based on half of the number of patients that was initially established. This change in terms of sample size, provided that alpha is set to 0.05 (one tail), will result in a reduction of the power of the study, which will pass from 80% to 56%.

All patients will be analysed according both intention-to-treat and per protocol principle. HR for DFS and OS will be estimated according to the Cox model and their relative 90% confidence intervals will also be reported. We will also provide estimates and confidence intervals for the crude hazards and cumulative incidence curves in either treatment arm.

LIST OF ABBREVIATIONS AND DEFINITION OF TERMS

The following abbreviations and special terms are used in this study protocol.

Abbreviation or special term	Explanation
AE	Adverse event
ALP	Alkaline phosphatase
ALT	Alanine aminotransferase
ANC	Absolute neutrophil count
Assessment	An observation made on a variable involving a subjective judgement (assessment)
AST	Aspartate aminotransferase
BUN	Blood urea nitrogen
CI	Confidence interval
CHF	Congestive Heart Failure
CRF	Case report form
CT	Computerised tomography
CTC	Common toxicity criteria
DQS	Data query sheet
ECG	Electrocardiogram
EDTA	Ethylene diamine tetra-acetic acid
ER	Oestrogen receptor
GCP	Good Clinical Practice
GI	Gastrointestinal
HDPE	High density polyethylene
HIV	Human immunodeficiency virus
IB	Investigator's Brochure
IEC	Independent Ethics Committee
IHC	Immunohistochemistry
INR	International Normalised Ratio
ITT	Intention-to-treat
iv	Intravenous
L/l	Litre
LLT	Low level term
mg	Milligram
ml	Millilitre
MUGA	Multigated acquisition
NCI	National Cancer Institute
NYHA	New York Heart Association
Measurement	An observation made on a variable using a measurement device.
Outcome variable	A variable (usually a derived variable) specifically defined to be used in the analysis of a study objective.
Parameter	A quantity (usually unknown) that characterises the distribution of a variable in a population of subjects.
PgR	Progesterone receptor
Principal investigator	A person responsible for the conduct of a clinical study at an investigational study site. Every investigational study site has a principal investigator.
PS	Performance status
PT	Prothrombin time
SAE	Serious adverse event
SAP	Statistical Analysis Plan
SI Unit	Standard International Unit
SRO	Subject-reported outcome
ULN	Upper limit of normal
Variable	A characteristic or a property of a subject that may vary e.g. from time to time or between subjects
WHO	World Health Organisation

1 BACKGROUND AND RATIONALE

About 20% of breast cancer shows HER2-receptor over-expression, that can be measured by immunohistochemistry or gene amplification. HER2 overexpressing tumors have aggressive biological characteristics (high proliferative activity, metastatic potential and neoangiogenesis) and poor survival (median about 3 years vs 6-7 years for HER2-negative tumors).
Trastuzumab (Herceptin®, in the following, H) is a humanized monoclonal antibody against HER2 receptor. The linkage of H with the receptor blocks its activation, which in turn induces an arrest in the downstream intracellular transduction pathway and prevents the transcription of related genes. In vitro, H demonstrated synergism with several cytotoxics in particular anthracyclines, taxanes, platinum salts and vinorelbine. The combination of H and chemotherapy induces increased cytotoxicity, with increased apoptosis and decreased cell proliferation.
The cytotoxic synergism of combined H and chemotherapy is also supported by clinical data in metastatic and neoadjuvant settings. In HER2 + advanced disease, the combination of H and chemotherapy resulted in 25% survival increase (from 20 to 25 months median survival). The two pivotal studies with paclitaxel and docetaxel respectively have also demonstrated that patients receiving the H and chemotherapy combination upfront had a better outcome than those who received H at disease progression only. These observations further support the synergistic cytotoxic effects of an early combination of H and chemotherapy.
Similar conclusions can be drawn from neoadjuvant studies. At MDACC, patients with HER2 positive operable breast cancer received upfront chemotherapy (4 courses of paclitaxel followed by 4 courses of FEC - fluorouracil, epidoxorubicin and cyclophosphamide) with or without concomitant H. The rate of pathological complete response (pCR) was 26.3% without and 66.7% with H respectively.
Finally, a large benefit in disease free survival with promising survival gain has been reported from 5 different randomized studies with H in the adjuvant setting. These trials have included more than 10,000 women with HER2 positive breast cancer. In these trials, one year of H reduced by 50% the risk of relapse (HR 0.48 in the combined analysis of the NSABP-B31 e NCCTG N9831 studies; 0.54 in the HERA study; 0.61 in the AC-TH arm and 0.67 in the TCH arm of the BCIRG006 trial). Moreover, at an interim analysis of N9831 trial, even if with a limited number of events, an advantage for the combination of chemotherapy and H over the sequence has been reported (HR 0.64 in favour of H initiated concomitantly with paclitaxel versus H following by paclitaxel).
In conclusion, both biological and clinical data strongly support the synergistic cytotoxic effects of H and chemotherapy on HER2 positive breast cancer cells, while the sequential administration of H after chemotherapy seems to induce mainly a cytostatic effect that might require longer treatment to achieve maximum clinical benefit.
Unfortunately, the only study prospectically designed to test different durations of H administration is the HERA trial; at present however, the results of the comparison of one versus two years of treatment are not yet available. By now, on the basis of the results of the American and European studies, one year of treatment with H can be considered the gold standard.
However, in a small adjuvant study from Finland, H has been given upfront for 3 months (9 weeks) in combination with Docetaxel or Vinorelbine followed by 3 FEC courses without H. At

a median follow-up of 36 months, the reduction in the risk of relapse is similar (HR 0.42) to that observed in the previous studies with 1 year of H.
Interestingly, very recently, ECOG has presented the results of E2198 trial that reinforces the observation coming from Finland. This pilot trial was designed to explore the cardiac safety of short versus more prolonged adjuvant H and was not powered to compare treatment efficacy. 157 HER2 positive patients were randomized to 10 weeks of H plus paclitaxel followed by AC, or to the same chemotherapy regimen followed by H administered up to one year after chemotherapy. At 5 years, disease free survival was 78% and 81%, and overall survival was 91% and 86% in the short and long H treatment arm, respectively.
Finally, in the neoadjuvant study from MDACC already mentioned, where H was given for 6 months concomitantly with chemotherapy, at a median follow-up of 2 years, no relapses have been observed.
These data confirm that optimum adjuvant H duration remains to be established and suggest that shorter treatment durations might produce comparable efficacy with significant lower toxicities and costs.
In particular, the unexpected 13% rate of severe cardiac events reported with H in advanced disease, has raised concern on the cardiac safety of H plus chemotherapy for the treatment of early breast cancer. As a matter of fact, H by itself is not directly cardiotoxic, but, by inhibiting the survival pathway activated through HER2 receptors, can impair the reparative mechanisms activated by myocardial cells following oxidative stress induced by several agents including cytotoxics (in particular anthracyclines).
Cardiac safety has been closely evaluated and monitored in H adjuvant studies. In all the studies, prior cardiac morbidities including uncontrolled angina and severe hypertension were exclusion criteria; moreover , the patients had to have a normal left ventricular ejection fraction (LVEF) before starting H. Despite these selection criteria, in NSABP B31, 6.7% of the patient did not start H due to a >16% reduction in LVEF after anthracyclines. Among the 1159 patients who started H, 31.4% stopped the treatment before the 52 planned weeks: main reasons for stopping were an asymptomatic (14.2%) or symptomatic (4.7%) decline in the LVEF. The cumulative incidence of NYHA grade III or IV congestive heart failure (CHF) was 4.1% in patients treated with chemotherapy plus H versus 0.8% in those treated with chemotherapy alone. Fortunately, only 1 out the 31 patients with CHF had symptoms lasting more than 6 months.
In the N9831 trial, the cumulative incidence of CHF was 3.5% and 2.6% in patients randomized to concomitant or sequential H, respectively, versus 0.2% in the control arm. The cardiac toxicity was reported early in patients receiving concomitant H and chemotherapy, whereas showed a progressive increase in patients who had H after termination of chemotherapy.
In the HERA trial, H was stopped in 8.5% of the patients. A symptomatic CHF was recorded in 1.7% of the patients who received H, 2 versus 0.06% in the control arm. An asymptomatic decline in EF was observed in 7.1% of the patients treated with H, versus 2.2% of the controls.
The different incidence of cardiac toxicities reported in the American trials and in the HERA study can be partly related to the different definition of cardiac event: a decline in LVEF >15%, or between 10 and 15% if below the normal limits, in the American studies; > 10% decline from basal or below 50% in the HERA study.
In the BCIRG 006 trial, the incidence of grade III-IV cardiac events was 1.87% among patients treated with AC-TH, 0.38% among patients who received H without anthracyclines,

and 0.38% in the control arm. The decline of EF >10% was 18%, 8.6% and 10% in the three arms, respectively.
In the Finland study with short H treatment, no severe cardiac toxicity has been reported, while 3.5% of the patients showed a transient decline of EF >15%.
In summary:
1) preclinical and clinical data (in metastatic, adjuvant and neoadjuvant settings) support the concept that early combination of H with chemotherapy induces a synergistic cytotoxic effect;
2) adjuvant H treatment reduces by about 50% the risk of relapse and increases overall survival;
3) the concomitant administration of H with chemotherapy seems more effective than the sequential use of either treatments;
4) the risk of CHF is about 4% when H is administered concomitantly to chemotherapy and about 2% if given sequentially;
5) the percent of patients that, for any reasons (mainly CHF or decline in LVEF), stop or do not start H treatment is about 18% in the HERA trial, 35% in the combined analysis of B31 and N9831, and 10% in the Finland study;
6) despite significant differences in treatment duration and rate of interruptions/patients never treated, all these studies showed similar efficacy (HR for DFS around 0.50).
Finally, even if pharmacoeconomics is not an end point of this proposal, the economic impact of the adjuvant H treatment must also be considered. In Italy, 35,000 new breast cancers are diagnosed each year. Assuming the criteria adopted by the HERA trial and recommended by AIFA to select patients for adjuvant H , and limiting the treatment to women below 75 years without important cardiac comorbidities, we can estimate that , each year, 2500-3000 patients will require H. The median cost of one year H is about 25,000 Euro/patient, while a short 9 weeks treatment would cost 4,400 Euro/patient. If equally efficacious and, possibly, less toxic, a short treatment according to the FinHer schema would therefore result in significant savings for the National Health System.
On the basis of these considerations, we have designed a prospective, randomized, non-inferiority study assessing the efficacy and cardiac tolerability of short vs long adjuvant H treatment combined with chemotherapy, in HER2 positive breast cancer patients.
Other ongoing/planned trials are addressing the question of shorter duration of Herceptin:
1) the PHARE trial sponsored by the Istitut National du Cancer in France will randomize about 4,000 patients to 6 versus 12 months of Herceptin
2) the SOLD trial sponsored by the Finnish Breast Cancer Group in Scandinavia, will randomize about 3,000 patients to 9 versus 52 weeks of Herceptin
3) the PERSEPHONE trial sponsored by NCRI in UK will randomize about 4,000 patients to 6 versus 12 months of Herceptin
Overall these three trials and our Shorther study, will include almost 14,000 patients with HER2+ disease randomized to shorter vs standard 1-yr herceptin. PIs of these trials have already discussed and agreed with the possibility of performing a meta-analysis of these studies.

2. STUDY OBJECTIVES

The primary objective is to evaluate if 3 months of Herceptin administered according to the Finnish protocol (9 weekly administrations) is not inferior to 12 months (18 three-weekly administrations) in a standard chemotherapy protocol in term of disease free survival, in patients with HER2 positive early breast cancer.

Second primary end point is overall survival. The study is designed assuming that prolonged H administration concomitantly with chemotherapy produces more frequent cardiac events than shorter H treatment, and that the onset of this potentially serious toxicity might preclude the benefit of treatment to a significant proportion of patients.

2.1 Primary objective

° Disease free survival (DFS), calculated as the time interval between randomization and any of the following events, whichever first: local, regional and distant recurrence; contralateral breast cancer, excluding in situ carcinoma; other second primary cancer; death before recurrence or second primary cancer. Patients who will not experience relapse at the time of the last follow-up will be censored.

° Overall survival (OS) will be evaluated as second primary analysis outcome. The survival will be calculated as the time-interval between randomization and patient death or last follow-up.

2.2 Secondary objectives

° Failure Rate (FR) at 2 years, calculated as cumulative incidence of relapse, contralateral breast cancer (excluding in situ carcinoma), death for all causes, treatment withdrawal due to toxicity of therapy.

° Incidence of cardiac events (defined as decrease of EF > 15% from basal values, or decrease > 10% with EF absolute value below 50%, or symptomatic cardiac failure, or other cardiac side effects grade 2 or more according to NCI CTCAE (version 3, published 31 March 2003)

3. STUDY PLAN AND PROCEDURES

3.1 Overall study design and flow chart

This is a multicenter, randomized phase III trial, in HER2 neu positive resected early breast cancer patients.

100 Italian Centres, coordinated by 8 Regional Centres will participate to this study.

Patients must have already completed the surgical treatment, consisting in the surgical resection of the tumor with free margins (i.e. no neoplastic cells on the resected margin) and treatment of the axilla (node negative sentinel node or complete axillary clearance). In case of micrometastasis in the sentinel node (between 0.2 and 2 mm) clearence of the axilla has to be done. In case of isolated tumor cells in the sentinel node, surgery of the axilla is not mandatory.

Interval from completion of surgical procedures and patient randomization must not exceed 10 weeks. Treatment must be started within 2 working days from randomization.

After the end of chemotherapy, in case of conservative surgery, or in case of > than 4 axillary-nodal involvement, patients will undergo to radiotherapy. The irradiated fields will be defined by radiotherapist according to the tumor size, and the number of positive axillary nodes.
Radiotherapy must be started within 8 weeks by the end of chemotherapy.
In patients enrolled in the long treatment arm, radiotherapy will be delivered concomitantly with trastuzumab.
Patients with hormone-receptor positive tumor (ER and/or PGR) will receive hormonal treatment after chemotherapy. Patients enrolled in the long treatment arm will receive hormonal therapy concomitantly with trastuzumab.
Premenopausal patients will be treated with monthly LHRH-agonist for 2 years plus daily tamoxifen for 5 years.
Post-menopausal patients will receive an aromatase inhibitor, daily for 5 years. The menopausal condition is defined by at least one of the following:

- 60 years of age
- < 60 years of age and amenorrheic for 12 months prior to day 1
- < 60 years of age and amenorrheic for < 12 months prior to day 1 with luteinizing hormone (LH) and follicle stimulating hormone (FSH) values within postmenopausal range, or without a uterus
- Prior bilateral oophorectomy
- Prior radiation castration with amenorrhea for at least 6 months

3.2 Study Procedures

Note: procedures, examinations, and laboratory assessments may be performed more frequently, if clinically indicated.
The flowcharts of the study are reported in table 1 (for ARM A) and in table 2 (for ARM B)

Within 12 weeks
- Full staging with: chest radiogram (all the patients), liver ultrasound (all the patients); bone scan (when clinically indicated or in case of clinically positive axillary nodes)

Within 28 days prior to first dose
- Informed consent form (ICF)
- Physical examination
- Medical history, ECOG Performance Status; baseline signs and symptoms
- Record all medication(s) received within 2 weeks prior to the first dose of investigational product and note if the medication is continuing
- Electrocardiogram (12-lead ECG)
- Vital signs (blood pressure and pulse rate body temperature, height, and body weight)
- Hematology and blood chemistry (see Laboratory Assessments), urinalysis
- Serum pregnancy test for women of childbearing potential.
- Echocardiogram or MUGA scan (note that baseline and on treatment scans should be performed using the same modality and preferably at the same institution).

Assessment prior to each treatment administration:
- Hematology and blood chemistry (see Laboratory Assessments)
- Physical examination
- ECOG Performance Status
- Vital signs (blood pressure and pulse rate, body temperature, and body weight)

- Record any non-serious and serious AEs (see Section 4.6) and assign appropriate toxicity grade (NCI CTCAE version 3, published 31 March 2003)
- Record all concomitant medication(s) added and/or changed

Cardiac evaluations

Echocardiogram or MUGA scan (note that baseline and on treatment scans should be performed using the same modality and preferably at the same institution) will be performed:
° ARM A: at the end of AC or EC chemotherapy, and subsequently at month 6, 9, 12, 18 since randomization; thereafter, cardiac evaluation will be performed yearly.
° ARM B: after docetaxel plus trastuzumab, and subsequently at month 6, 9, 12, 18 since randomization; thereafter, cardiac evaluation will be performed yearly.

Centralized revaluation of Her2 status

Tumor tissue sample will be sent to the center of Modena for the centralized revaluation of Her2 status and then stored c/o the Pathology Institute of Policlinico of Modena. Any further use of the biological material cited above in new research projects regarding breast cancer will be planned and performed in the coordinating center and will be subject to the approval of the Competent Ethical Committee. For this purpose the biological samples will be transferred from University of Modena to Oncologia Medica 2 - Dept. of Surgery, Oncology and Gastroenterology – University of Padova, Italy
.

Assessments at Study Conclusion or Withdrawal from Therapy

The following assessments will be performed when a subject is withdrawn from the study for any reason:

• Physical examination, including vital signs (blood pressure and pulse rate after 5 minutes sitting, body temperature, and body weight)
• ECOG Performance Status
• Electrocardiogram (12-lead ECG)
• Record any AE(s) and serious adverse event(s) (SAEs) and assign appropriate toxicity grade (NCI CTCAE version 3, published 31 March 2003)
• Record all concomitant medications(s) added and/or changed
• Hematology and blood chemistry (see Laboratory Assessments) will be performed if the last assessment was > 4 weeks from date of the last dose of investigational product
• Cardiac evaluation will be performed if the last assessment was > 8 weeks from the date of the last dose of investigational product

Follow-up

After randomization, follow up including clinical examination, complete blood chemistry, CEA, CA 15-3, chest radiogram and liver ultrasound will be performed every 6 months during the first five years and yearly thereafter. Bone scan will be repeated only when clinically indicated (pain, elevation of alkaline phosphatase and/or tumor markers). Mammography will be repeated every 12 months. Ecocardiography will be repeated yearly after month 18.
After 5 years, annual assessment of the patient status is required (dead or alive; with or without recurrence or second primary tumor) for overall survival evaluation.
Long-term follow-up will be attempted, whenever possible.

3.3 Laboratory assessment

The following parameters will be evaluated:

Biochemistry (serum gel tube- Sample volume 6-9 ml), Hematology (EDTA coated tubes- Sample volume 3 ml)
ALT, AST
Alkaline Phosphatase
Total Bilirubin
Blood Urea Nitrogen or urea
Creatinine
White blood cell count (total)
Absolute neutrophil count
Hemoglobin
Platelet count

3.4 Pregnancy Test

Pregnancy Test: a screening serum beta-hCG (human chorionic gonadotrophin) pregnancy test is mandatory for all women of childbearing potential within 2 weeks prior to the first dose of investigational product. Thereafter, the serum pregnancy test need only be repeated if clinically indicated or as required by local regulation

3.5 Serum ECD determination

Serum sample for ECD determination will be collected before starting adjuvant therapy and at the end of treatment.
All the ECD determination will be assessed by a reference laboratory, by ELISA.

Table 1: Flow chart ARM A (AC or EC followed by P or D + H followed by H)
° Echocardiography to be repeated at month 18 from starting treatment and yearly thereafter

	Screening		Treatment period AC or EC	Before Starting H	Treatment period T+H	After T+H	Treatment Period H	After H
**Day/Day of cycle/Week**	**- 12 w**	**- 4 to 0 w**	**Day 1**		**Day 1**			
Informed consent		X						
Medical history		X						
Demography		X						
Randomisation		X						
Pregnancy test (if appropriate) **(within 2 weeks)**		X						
Inclusion/exclusion criteria		X						
Physical examination (vital signs, performance status and weight)		X	X		X		X	X
Liver US, Chest XR	X					X	Every 6 months to randomization	X
Bone Scan (when clinically indicated or in case of positive axillary nodes)	X							
Histological diagnosis (Samples of tumor tissue sent to Modena)		X						
Haematology		X	X		X			X
Biochemistry		X	X		X			X
Urinalysis		X						X
Serum ECD determination		X						X
Electrocardiogram (ECG)		X		X		X	X	
ECHO (MUGA)		X		X		X	Every 3 months	X°
Adverse events			X		X		X	X
Trastuzumab administration					X		X	
Chemotherapy administration			X		X			

Table 2: Flow chart ARM B (D+H followed by FEC)
° Echocardiography to be repeated at month 9, 12 and 18 from starting therapy and yearly thereafter

	Screening		Treatment period D+H	After D+H	Treatment period FEC	After FEC
**Day/Day of cycle/Week**	**-12 w**	**- 4 to 0 w**	**Day 1**		**Day 1**	
Informed consent		X				
Medical history		X				
Demography		X				
Randomisation		X				
Pregnancy test (if appropriate) **(within 2 weeks)**		X				
Inclusion/exclusion criteria		X				
Physical examination (vital signs, performance status and weight)		X	X		X	X
Liver US, Chest XR	X					Every 6 months to randomization
Bone Scan (when clinically indicated or in case of positive axillary nodes)	X					
Histological diagnosis (Samples of tumor tissue sent to Modena)		X				
Haematology		X	X		X	X
Biochemistry		X	X		X	X
Urinalysis		X				X
Serum ECD determination		X				X
Electrocardiogram (ECG)		X		X		X
ECHO (MUGA)		X		X		**X°**
Adverse events			X		X	X
Trastuzumab administration			X			
Chemotherapy administration			X		X	

3.6 Selection of study population

3.6.1 Study selection record

Investigators must keep a record of subjects who were considered for enrolment but were never enrolled. This information is necessary to establish that the subject population was selected without bias.

3.6.2 Inclusion criteria

Patients are eligible if they meet the following criteria:
- Surgically resected infiltrating primary breast cancer, HER2 positive tumor (either IHC 3+ or FISH+ according ASCO guidelines i.e. > 2.2; in case of polisomy, with $\geq$ 6 gene copies) , suitable for adjuvant chemotherapy
-Adequate treatment of axillary lymphnodes (SNB or ALND)
- Node positivity, or node negativity AND at least ONE of the following: T > 2 cm, Grade 3, presence of lymphovascular invasion, Ki 67> 20%, age < 35, hormone receptor negativity (<10%)
- Age >18, 75 years - ECOG PS 0-1
- Normal liver, renal and marrow function, defined AS: leukocytes >3000/mcL, absolute neutrophil count >1,500/mcL, platelets >100,000/mcL, total bilirubin within normal Institutional limits, AST (SGOT)/ALT(SGPT) 2.5Xinstitutional upper limit of normal, Creatinine within normal institutional limits
- Cardiac ejection fraction within the institutional range of normal as measured by echocardiogram or MUGA scan
- Patients should start treatment within 10 weeks from the date of surgery
- Women of child-bearing potential must agree to use adequate contraception (hormonal or barrier method of birth control or abstinence) prior to study entry and for the duration of study participation. Should a woman become pregnant or suspect she is pregnant while participating in this study, she should inform her treating physician immediately, the patient should be apprised of the potential hazard to the fetus and potential risk for loss of the pregnancy
- Ability to understand and the willingness to sign a written informed consent document
- Patients who have been treated for other infiltrating tumors, including breast cancer, and are disease free since at least 10 years, are eligible to the study

3.6.3 Exclusion criteria

Patients will be excluded from the study for any of the following reasons
- Stage IIIB or IV breast cancer
- More than 10 weeks from surgery (for patients undergoing re-excision of positive margins, or ALND following positive sentinel node biopsy, date of the last surgery will be taken in consideration)
- Contraindication to the treatment with anthracycline, cyclophosphamide, 5FU, paclitaxel, or trastuzumab
- prior treatment with chemotherapy, endocrine therapy or radiotherapy, with the exception of the patients treated with curative intent for other tumors, including breast cancer, since 10 years or more.
- Treatment with any other investigational agents

- Uncontrolled inter-current illness including, but not limited to, ongoing or active infection, symptomatic congestive heart failure, unstable angina pectoris, cardiac arrhythmia, or psychiatric illness/social situations that would limit compliance with study
requirements
- Pregnancy or breastfeeding (breast feeding should be discontinued to be enrolled in the study)
- Women of childbearing potential that refusal to adopt adequate contraceptive measures

3.6.4 Restrictions

Women of child-bearing potential must be willing to practice acceptable methods of birth control to prevent pregnancy (contraceptive measures must be taken for at least three months following the discontinuation of therapy).

3.7 Discontinuation of subjects from treatment and assessment

3.7.1 Criteria for discontinuation

Patients may be discontinued from study treatment and assessment at any time. Specific reasons for discontinuing a subject from this study are:
1. Voluntary discontinuation by the subjects who are at any time free to discontinue their participation in the study, without prejudice to further therapy
2. Safety reasons as judged by the investigators
3. Severe non compliance to treatment, as judged by the Investigators
4. Incorrect enrolment or randomization of the subject.
5. Death
6. Subject lost to follow up
7. Objective progression of the disease

3.7.2 Procedures for discontinuation

The reason for discontinuation and the date must be documented on the case report form (CRF).
At discontinuation, all on-going study related toxicities and SAEs must be followed until resolution, unless in the investigators' opinion the condition is unlikely to resolve, due to the subject's underlying disease.
After discontinuation from treatment, subjects must be followed up for all the existing and new AEs for 30 days after the last dose of study drug. All new AEs occurring during that period must be recorded and followed up until resolution.

3.8 Treatments

3.8.1 Investigational products

All chemotherapy drugs used in the protocol, both in control and experimental arm, are licensed with the indication of adjuvant treatment of breast cancer.
Trastuzumab is licensed for adjuvant treatment of HER2 positive breast cancer, both sequential and concurrent with chemotherapy.
The drugs used in the study are commercially available, and will not be reimbursed.

3.8.2 Doses and treatment regimens

Patients will be randomized to receive:
- Treatment Arm A (Long): AC or EC x 4 courses followed by paclitaxel (or docetaxel) plus concomitant trastuzumab q 3 wks x 4 courses followed by trastuzumab for 14 additional doses every 3 weeks (total 18 3-wkly doses).
- Treatment Arm B (Short): Docetaxel for 3 courses plus trastuzumab weekly for 9 weeks followed by FEC times 3 (total 9 wkly doses).

3.8.3 Treatment details

Arm A

Investigators should administer either AC (adriamycin 60 mg/sqm plus cyclophosphamide 600 mg/sqm) or EC (epidoxorubicin 90 mg/sqm plus cyclophosphamide 600 mg/sqm) i.v infusion on day 1 every 21 days for 4 courses.

After 4 courses of AC or EC, patients will receive trastuzumab iv (8mg/kg loading dose starting with the 1st cycle of taxane, and 6 mg/kg thereafter) for 4 courses every 21 days and docetaxel or paclitaxel as follows:

- patients < 65 years: **docetaxel 100 mg/sqm** iv infusion over 1 hour with standard premedication **or paclitaxel 175 mg/sqm** iv infusion over 3 hours with standard premedication

- patients ≥ 65 years: **docetaxel 80 mg/sqm** iv infusion over 1 hour with standard premedication **or paclitaxel 175 mg/sqm** (**dose unchanged for paclitaxel**) iv infusion over 3 hours with standard premedication

At completion of the chemotherapy program, patients will receive trastuzumab monotherapy 6 mg/kg iv infusion every 21 days for 14 additional courses (for a total of 18 trastuzumab doses).

Arm B

- **Patients < 65 years** : **Docetaxel 100 mg/ sqm** iv on day 1 every 21 days for 3 courses plus trastuzumab (4 mg/kg loading dose at 1st administration only, followed by 2 mg/kg) weekly for 9 weeks starting together with the first dose of docetaxel.
- **Patients ≥ 65 years old**: **Docetaxel 80 mg/sqm** iv on day 1 every 21 days for 3 courses plus trastuzumab (4 mg/kg loading dose at 1st administration only, followed by 2 mg/kg) weekly for 9 weeks starting together with the first dose of docetaxel

At completion of docetaxel-trastuzumab, patients will receive 3 courses of FEC. The FEC regimen (5-Fluorouracil 600 mg/smq, Epidoxorubicin 60 mg/sqm, Cyclophospamide 600 mg/sqm, iv on day 1) will be started 21 days after the last docetaxel administration, and it will be delivered every 21 days for 3 courses.

3.8.4 Prophylactic use of Colony stimulating factors (G-CSF)

Patients ≥ 65 yrs receiving Docetaxel (80 mg/sqm) can receive prophylactic G-CSF from the first course at the discretion of treating physician.

3.8.5 Storage

Trastuzumab should be stored at 2°C-8°C in a refrigerator.

3.8.6 Method of assigning subjects to treatment group

As eligibility is confirmed, patients will be randomized strictly sequentially, according to a randomization scheme prepared by the Biostatistician in the Coordinating Centre in Modena. The randomization will be performed on line. Patients will be stratified
according to nodal status, hormone-receptor status and Regional Coordinating Center. Patients will be identified by patient's initials, the date of birth and centre code. The investigator will receive the patient randomization number with treatment allocation
directly on line. The treatment administration must begin within 72 hours from the date of randomization. In case of discontinuation from the study, the randomization number will not be reused.

3.9 Toxicity

All drugs are well known and routinely used in the treatment of breast cancer. No unpredictable toxicity is expected from the treatment. However, both predefined and not predefined side effects will be recorded, classified, graded and managed according to NCI CTCAE (version 3, published 31 March 2003).

3.9.1 Cardiac toxicity

Because of the known effects of trastuzumab on cardiac function, only patients with normal LVEF are eligible for study and patients will undergo monitoring of LVEF during the study (at baseline and every 12 weeks thereafter). **Any patient who develops clinical signs or symptoms suspicious of cardiac failure should undergo an LVEF assessment**.
The majority of patients who develop heart failure improved with the standard medical treatment. This included: diuretics, cardiac glycosides, and/or angiotensin-converting enzyme (ACE) inhibitors.
Asymptomatic Events: rules for treatment discontinuation
Cardiac safety management for all ARMS:

1) same definition of cardiac dysfunction in the two arms
2) Same management of treatment discontinuation/restart in the two arms

<table>
<tr><th>Relationship of LVEF to LLN (lower limit of the normal)</th><th>Absolute decrease of < 10%</th><th>Absolute decrease of 10-15%</th><th>Absolute decrease of ≥ 16%</th></tr>
<tr><td>Within the Normal Limits</td><td>Continue</td><td>Continue</td><td>Hold*</td></tr>
<tr><td>1-5% below LLN</td><td>Continue</td><td>Hold*</td><td>Hold*</td></tr>
<tr><td>>/= 6% below LLN</td><td>Continue (reassess)§</td><td>Hold*</td><td>Hold*</td></tr>
<tr><td colspan="4">§: reassessment after 4 weeks (and every 4 weeks thereafter)

*ARM A and B (chemotherapy + trastuzumab): stop trastuzumab,.
1. Reassess after 4 weeks. If criteria for starting/continuation met: start/resume treatment.
2. Reassess after 4 weeks. If criteria for starting/continuation unmet: reassessment after 4 additional weeks; If criteria for starting/continuation met: start/resume treatment.
3. If 2 consecutive hold: hold/discontinue trastuzumab permanently.

NB: In case of restart of treatment after any starting delay/discontinuation due to LVEF ABNORMALITIES, LVEF should be assessed every 4 weeks.</td></tr>
</table>

Assessment pre-Trastuzumab/taxane treatment (ARM A)

In case of LVEF below LLN prior to start trastuzumab plus taxane regimen (at the end of 4 courses of AC or EC), the patients will receive only taxane, and will be re-assessed and managed according to the rules reported above.

Assessment pre-FEC Chemotherapy (ARM B)

In case of LVEF below LLN prior to start FEC regimen (at the end of 9 weeks of weekly trastuzumab plus 3-weekly docetaxel), the patients will receive subsequent treatment at treating physician's discretion.

Symptomatic cardiac events
Subjects with an NCI CTCAE Grade 3 or 4 left ventricular systolic dysfunction must be withdrawn from therapy.

A > 16% absolute decrease from baseline in LVEF (asymptomatic or symptomatic), that is below the institution's lower limit of normal is considered a SAE.

3.9.2 Chemotherapy dose reduction/interruption

Before each administration of chemotherapy, hematological parameters and biochemistry have to be controlled.
Full dosage of chemotherapy drugs will be delivered if WBC is □3 X 10^9/L, ANC is □1.5 □ 10^9/L and platelets □100 □ 10^9/L, and the patient has recovered below grade 2 for all non hematological toxicity.
A one week delay will be adopted for lower values, until a maximum of 3 weeks.
In case of grade 4 hematological toxicity the following dose adjustments are adopted:

Patients < 65 y old:
STEP 1 - In case of grade 4 hematological toxicity lasting more than 3 (ANC) or 5 (PLT) days or febrile neutropenia, all subsequent courses will be delivered **AT THE SAME DOSE with prophylactic G-CSF.**
STEP 2 - If, in spite of G-CSF, a grade 4 hematological toxicity or febrile neutropenia will occurred, a **dose reduction to Paclitaxel 150 mg/sqm or Docetaxel 80 mg/sqm** must be adopted, with optional G-CSF

Patients $\geq$ 65 y old:
Patients treated with Docetaxel - In case the patient **has not received** prophylactic use of G-CSF in the first course and a grade 4 hematological toxicity lasting more than 3 (ANC) or 5 (PLT) days or febrile neutropenia occurs, subsequent courses will be delivered at the **same dosage (docetaxel 80 mg/sqm) with prophylactic G–CSF.**
In case the patient **has received** prophylactic use of G-CSF and a grade 4 hematological toxicity lasting more than 3 (ANC) or 5 (PLT) days or febrile neutropenia occurs, **a dose**

reduction to Docetaxel 60 mg/sqm must be adopted, with optional G-CSF, for the subsequent courses.
Patients treated with Paclitaxel -In case of grade 4 hematological toxicity lasting more than 3 (ANC) or 5 (PLT) days or febrile neutropenia, subsequent courses will be administered with **paclitaxel 175 mg/sqm with prophylactic G-CSF**
If, in spite of prophylactic G-CSF, a grade 4 hematological toxicity lasting more than 3 (ANC) or 5 (PLT) days or febrile neutropenia occurs, , **a dose reduction to Paclitaxel 150 mg/sqm must be adopted, with optional G-CSF, for the subsequent courses.**

	1st course	1st Course after G4 neutropenia or febrile neutropenia		Subsequent course in case of further G4 neutropenia or febrile neutropenia
**< 65 years**				
Docetaxel	100 mg/sqm	100 mg/sqm + G-CSF		80 mg/sqm ± G-CSF
Paclitaxel	175 mg/sqm	175 mg/sqm + G-CSF		150 mg/sqm ± G-CSF
**≥ 65 years**		No proph. G-CSF	Proph. G-CSF	
Docetaxel	80 mg/sqm ± G-CSF	80 mg/sqm + G-CSF	60 mg/sqm ± G-CSF	60 mg/sqm + G-CSF
Paclitaxel	175 mg/sqm	175 mg/sqm + G-CSF	N.A.	150 mg/sqm + G-CSF

In case of sensory-motor neurological toxicity ≥ Grade 2, taxanes will be discontinued until recovery.
Chemotherapy will be permanently discontinued in case of non hematological toxicity grade 4, or in case of SAE. For grade 3 non-hematological toxicity, a case by case evaluation has to be made by the investigator.
Doses which have been reduced for toxicity must not be re-escalated
In case of allergic reactions, the taxane will be substituted with Vinorelbine (25 mg/sqm day 1and 8 every 21 days) in the subsequent courses.

3.9.3 Trastuzumab dose interruption/delayed

For trastuzumab there is no dose adjustment foreseen. The infusions will be stopped completely according to the following:

- **Development of NYHA class III/IV cardiac dysfunction**
- **Grade 3-4 non hematologic toxicity, until recovery to grade ≤2**

For asymptomatic patients with decrease of reduction the LVEF, refer to section 3.8.3
In case chemotherapy is delayed, trastuzumab will be
1) **delayed** and administered with the next chemotherapy course in Arm A (3 wly schedule)
2) **not delayed** in Arm B (wkly schedule)

3.10 Study Closure

Study closure will occur when the provided number of event will be reached. Complete follow-up of the patients will be continued until 5 years. Patients will be thereafter evaluated for survival.

4. MEASUREMENTS OF STUDY VARIABLES AND DEFINITIONS OF OUTCOME VARIABLES

4.1 Disease free survival

Disease free survival (DFS), calculated as the time interval between randomization and any of the following events, whichever first: local, regional and distant recurrence; contralateral breast cancer, excluding in situ carcinoma; other second primary cancer; death before recurrence or second primary cancer. Survival time of patients who have not experienced any event at the time of the last follow-up will censored. In order to avoid some of the problems related to non random censoring the backdating technique will be used, by selecting a closing date at 1 year before the conclusion of data collection. All survival times of patients disease-free on that date will censored at that time, and all events occurred after the closing date will be ignored in the analysis.

4.2 Overall survival

Overall survival (OS) will be evaluated as second primary analysis outcome. The survival will be calculated as the time-interval between randomization and patient death or last follow-up. The same backdating technique described above will be used.

4.3 Failure Rate

Failure rate (FR) at 2 years, calculated as cumulative incidence of relapse, contralateral breast cancer (excluding in situ carcinoma), death for all causes, treatment withdrawal due to toxicity of therapy.

4.4 Incidence of cardiac events

Incidence of cardiac events (defined as decrease of EF > 15% from basal values, or decrease > 10% with EF absolute value below 50%, or symptomatic cardiac failure, or other cardiac side effects grade 2 or more according to NCI CTCAE (version 3, published 31 March 2003)

4.5 Safety Profile

To assess the safety profile, nature, incidence and severity of adverse events (AEs) and serious adverse events (SAEs) will be collected. Incidence of and reasons for study drug dose interruption or reduction and discontinuation will be collected. Toxicities will be graded using NCI Common Terminology Criteria for Adverse Events (CTCAE) version 3.0.

4.5.1 Adverse Events (AE) and serious Adverse events (SAE)

The investigator is responsible for the detection and documentation of events meeting the criteria and definition of an AE or SAE, as provided in this protocol. During the study when there is a safety evaluation, the Investigator or site staff will be responsible for detecting, documenting and reporting AEs and SAEs, as detailed in both this section of the protocol and in the AE/SAE section of the CRF

4.5.1.1 Definition of an AE

Any untoward medical occurrence in a patient or clinical investigation subject, temporally associated with the use of a medicinal product, whether or not considered related to the medicinal product.
An AE can therefore be any unfavourable and unintended sign (including an abnormal laboratory finding), symptom, or disease (new or exacerbated) temporally associated with the use of a medicinal product. For marketed medicinal products, this also includes failure to produce expected benefits (i.e. lack of efficacy), abuse or misuse.
Examples of an AE include:
• Significant or unexpected worsening or exacerbation of the condition/indication under study.
• Exacerbation of a chronic or intermittent pre-existing condition including either an increase in frequency and/or intensity of the condition.
• New conditions detected or diagnosed after investigational product administration even though it may have been present prior to the start of the study.
• Signs, symptoms, or the clinical sequelae of a suspected interaction.
• Signs, symptoms, or the clinical sequelae of a suspected overdose of either investigational product or a concurrent medication (overdose per se should not be reported as an AE/SAE).
Examples of an AE do not include a/an:
• Medical or surgical procedure (e.g., endoscopy, appendectomy); the condition that leads to the procedure is an AE.
• Situations where an untoward medical occurrence did not occur (social and/or convenience admission to a hospital).
• Anticipated day-to-day fluctuations of pre-existing disease(s) or condition(s) present or detected at the start of the study that do not worsen.
• The disease/disorder being studied, or expected progression, signs, or symptoms of the disease/disorder being studied, unless more severe than expected for the subject’s condition.

4.5.1.2 Definition of a SAE
A serious adverse event is any untoward medical occurrence that, at any dose:
a) results in death.
b) is life-threatening.
NOTE: The term 'life-threatening' in the definition of 'serious' refers to an event in which the subject was at risk of death at the time of the event. It does not refer to an event, which hypothetically might have caused death, if it were more severe.
c) requires hospitalization or prolongation of existing hospitalization.
NOTE: In general, hospitalization signifies that the subject has been detained (usually involving at least an overnight stay) at the hospital or emergency ward for observation and/or treatment that would not have been appropriate in the physician’s office or out-patient setting. Complications that occur during hospitalization are AEs. If a complication prolongs hospitalization or fulfils any other serious criteria, the event is serious. When in doubt as to whether “hospitalization” occurred or was necessary, the AE should be considered serious.
Hospitalization for elective treatment of a pre-existing condition that did not worsen from baseline is not considered an AE.
d) results in disability/incapacity
NOTE: The term disability means a substantial disruption of a person’s ability to conduct normal life functions. This definition is not intended to include experiences of relatively minor medical significance such as uncomplicated headache, nausea, vomiting, diarrhea, influenza, and accidental trauma (e.g. sprained ankle) which may interfere or prevent everyday life functions but do not constitute a substantial disruption.

e) is a congenital anomaly/birth defect.
Medical or scientific judgement should be exercised in deciding whether reporting is appropriate in other situations, such as important medical events that may not be immediately life-threatening or result in death or hospitalization but may jeopardize the subject or may require medical or surgical intervention to prevent one of the other outcomes listed in the above definition. These should also be considered serious. Examples of such events are invasive or malignant cancers, intensive treatment in an emergency room or at home for allergic bronchospasm, blood dyscrasias or convulsions that do not result in hospitalization, or development of drug dependency or drug abuse.

Additional protocol defined criteria:
• Cardiovascular events have been seen in subjects taking other compounds that inhibit ErbB2 when used in combination with or following anthracyclines and or taxane.As a precaution, the following will be reported as SAE:
• Cardiac dysfunction will be reported as an SAE and will be defined as any signs or symptoms of deterioration in LVEF that are Grade 3 (NCI CTCAE) or a 16% decrease in LVEF from baseline, and cardiac ejection fraction is below the institution's lower limit of normal. Refer to NCI CTCAE grading of left ventricular cardiac function.
Subjects with an NCI CTCAE Grade 3 or 4 left ventricular systolic dysfunction must be withdrawn from treatment.

4.5.2 Disease-Related Events or Outcomes Not Qualifying as SAEs
An event which is part of the natural course of the disease under study (e.g., disease progression) does not need to be reported as SAE. Progression of the subject's neoplasia will be recorded in the clinical assessments in the CRF. Death due to progressive disease is to be recorded on the 'Record of Death' CRF page and not as SAE. However, if the progression of the underlying disease is greater than that which would normally be expected for the subject, or if the investigator considers that there was a causal relationship between treatment with randomized therapy or protocol design/procedures and the disease progression, then this must be reported as SAE. Any new primary cancer must be reported as SAE.

4.5.3 Lack of Efficacy
“Lack of efficacy” per se will not be reported as an AE. The signs and symptoms or clinical sequelae resulting from lack of efficacy will be reported if they fulfill the AE or SAE definition (including clarifications).

4.5.4 Clinical Laboratory Abnormalities and Other Abnormal
Assessments as AEs and SAEs
Abnormal laboratory findings (e.g., clinical chemistry, haematology, urinalysis) or other abnormal assessments (e.g., echocardiogram, MUGA scan) that are judged by the investigator **as clinically significant** will be recorded as AEs or SAEs if they meet the definition of AE, or SAE, as defined in the relevant sections. Clinically significant abnormal laboratory findings or other abnormal assessments that are detected during the study or are present at baseline and significantly worsen following the start of the study will be reported as AEs or SAEs. However, clinically significant abnormal laboratory findings or other abnormal assessments that are associated with the disease being studied, unless judged by the investigator as more severe than expected for the subject’s condition, or that are present or detected at the start of the study and do not worsen, will not be reported as AEs or SAEs.

The investigator will exercise his or her medical and scientific judgement in deciding whether an abnormal laboratory finding or other abnormal assessment is clinically significant.

4.6 Time Period, Frequency, and Method of Detecting AEs and SAEs

From the time a patient consents to participate in the study until she has completed the study (including any follow-up period), all SAEs assessed as related to study participation (e.g., protocol-mandated procedures, invasive tests, or change in existing therapy), will be reported promptly to National Coordinating Centre.
All AEs and SAEs regardless of relationship to randomized therapy will be collected from the first dose of randomized therapy to 30 days after the last dose of randomized therapy and recorded on the CRF.

SAEs brought to the attention of the investigator at any time after cessation of randomized therapy and considered by the investigator to be related or possibly related to randomized therapy must be reported if and when they occur. Additionally, in order to fulfill international reporting obligations, SAEs that are related to study participation (e.g., procedures, invasive tests, change from existing therapy) or are related to a concurrent medication will be collected and recorded from the time the subject consents to participate in the study until he/she is discharged.
Subjects will be monitored at each scheduled assessment at the site (approximately every 4 weeks), at any contact with the subject during the study, and at the withdrawal visit, for the occurrence of AEs/SAEs. The investigator or designee will inquire about the occurrence of AEs/SAEs at every visit/contact during the study and throughout the 30 day follow-up period by asking the following standard questions:
1. How are you feeling?
2. Have you had any (other) medical problems since your last visit?
3. Have you taken any new medications since your last visit/assessment?

4.6.1 Recording of AEs and SAEs

When an AE/SAE occurs, it is the responsibility of the investigator to review all documentation (e.g., hospital progress notes, laboratory, and diagnostics reports) relative to the event. The investigator will then record all relevant information regarding an AE or SAE on the CRF. It is not acceptable for the investigator to send photocopies of the subject’s medical records in lieu of completion of the appropriate AE or SAE CRF pages.
The investigator will attempt to establish a diagnosis of the event based on signs, symptoms, and/or other clinical information. In such cases, the diagnosis should be documented as the AE/SAE and not the individual signs/symptoms.
Any AEs or SAEs occurring during the study must be documented in the subject's medical records and on the appropriate page of the CRF. Each AE or SAE is to be recorded individually. Deaths due to progressive disease are to be recorded on the 'Record of Death' CRF page and not as an SAE.
AEs and subject-completed questionnaires are independent components of the study. Responses to each question in the questionnaires will be treated in accordance with standard scoring and statistical procedures detailed by the scale’s developer. The use of a single question from a multidimensional health survey to designate a cause-effect

relationship to an AE is inappropriate.

4.6.2 Evaluating AEs and SAEs

4.6.2.1 Assessment of Intensity

The investigator will make an assessment of intensity of each AE and SAE reported. In this protocol, the intensity of AEs and SAEs will be graded on a scale of 1 to 5 according the National Cancer Institute (NCI) Common Toxicity Criteria for Adverse Events (CTCAE) Version 3.0 and are available at http://ctep.nci.gov/reporting/ctc.html. For SAEs, the maximum intensity (or grade) will be reported in the CRFs. For non-serious AEs, each change in intensity (or grade) will be reported in the CRFs.

4.6.2.2. Assessment of Causality

The investigator is obligated to assess the relationship between the study medical product and the occurrence of each AE/SAE. The investigator will use clinical judgement to determine the relationship. Alternative causes, such as natural history of the underlying diseases, concomitant therapy, other risk factors, and the temporal relationship of the event to the study drugs will be considered and investigated.

There may be situations when an SAE has occurred and the investigator has minimal information to include in the initial report. However, it is very important that the investigator always make an assessment of causality for every event prior to transmission of the SAE form to National Coordinating Centre. The investigator may change his/her opinion of causality in light of follow-up information, amending the SAE CRF accordingly. The causality assessment is one of the criteria used when determining regulatory reporting requirements.

The investigator will provide the assessment of causality as per instructions on the SAE form in the Investigators File.

4.6.2.3 Follow-Up of AEs and SAEs

After the initial AE/SAE report, the investigator is required to proactively follow each subject and provide further information on the subject's condition

All AEs and SAEs documented at a previous visit/contact and are designated as ongoing, will be reviewed at subsequent visits/contacts.

AEs that are ongoing with a toxicity of Grade 3 or 4, or have a relationship to study drug that is suspected (Reasonable Possibility) will be queried for resolution at study conclusion and at approximately 30 days after the last dose of randomized therapy.

Once resolved, the appropriate AE/SAE CRF page(s) will be updated. The investigator will ensure that follow-up includes any supplemental investigations as may be indicated to elucidate the nature and/or causality of the AE or SAE. This may include additional laboratory tests or investigations, histopathological examinations, or consultation with other health care professionals.

National Coordinating Centre may request that the investigator perform or arrange for the conduct of supplemental measurements and/or evaluations to elucidate as fully as possible the nature and/or causality of the AE or SAE. The investigator is obligated to assist. If a subject dies during participation in the study or during a recognized follow-up period, national

coordinating centre will be provided with a copy of any post-mortem findings, including histopathology.
New or updated information will be recorded on the originally completed SAE form in the Investigator's File, with all changes signed and dated by the investigator. The copy of the updated SAE form should be resent to National Coordinating Centre within the time frames outlined in 4.6.6

4.7 Reporting of SAEs to National Coordinating Centre (NCC)

SAEs will be reported promptly to NCC as described in the table below, once the Investigator determines that the event meets the protocol definition of SAE.

4.7.1 Timeframes for Submitting SAE Reports to NCC

Any SAEs which occur at any time during the clinical study or within 30 days of receiving the last dose of randomized therapy, whether or not related to the randomized therapy, must be reported to NCC. Once an investigator becomes aware that an SAE has occurred in a study subject, she/he will report the information to NCC within 24 hours by fax +39 049 821 5706 using the SAE form included in the Investigator's File.
Follow up information on SAEs must also be reported by the investigator within the same time frames (see the table below).

	Initial SAE Reports		Follow-up Information on a Previously Reported SAE	
Type of SAE	Time Frame	Documents	Time Frame	Documents
All SAEs	24 hrs	"SAE" CRF pages	24 hrs	Updated "SAE" CRF pages

If a non serious AE becomes serious, this and other relevant follow up information must also be reported to NCC according to the timeframe outlined in the table.

The SAE form will always be completed as thoroughly as possible with all available details of the event, signed by the investigator (or designee), and forwarded to NCC within the designated time frames. If the investigator does not have all information regarding an SAE, he/she will not wait to receive additional information before notifying NCC of the event and completing the form. The form will be updated when additional information is received.
The investigator will always provide an assessment of causality at the time of the initial Report as described in 4.6.4. Facsimile transmission of the "SAE form is the preferred method to transmit this information to the project contact for SAE receipt.
In rare circumstances and in the absence of facsimile equipment, notification by telephone/mail is acceptable. Concurrently a copy of the "SAE form" will be sent by mail to NCC Initial notification via the telephone does not replace the need for the investigator to complete and sign the SAE form within the time frames outlined in the above table.

4.7.2 Regulatory Reporting Requirements For Adverse Events

The Principal Investigator at NCC has a legal responsibility to notify, as appropriate, both the local regulatory authority and other regulatory agencies about the safety of a product under clinical investigation. Prompt notification of SAEs by the investigator to the appropriate project contact for SAE receipt is essential so that legal obligations and ethical responsibilities towards the safety of other subjects are met.
The Principal Investigator at NCC, or responsible person according to local requirements, will comply with the applicable local regulatory requirements related to the reporting of SAEs to regulatory authorities and the Independent Ethics Committee (IEC).
In particular, all the Suspected Unexpected **Serious Adverse Reactions** (SUSARs) that occur while on treatment and within 30 days since the last investigational drug administration, and that have a suspected relationship to study drug (Reasonable Possibility) will be notified with **urgency procedure** to the local regulatory Agency (AIFA) and EC with the following timelines:
- SUSARs that are considered life-threatening: notification within 7 days
-SUSARs that are not considered life-threatening: notification within 15 days.
The notification with urgency procedure is not required for SAEs that are expected with the drugs used in the protocol, and for non serious AEs, both expected or unexpected.
For these events (expected SAEs and AEs), the NCC will notify local regulatory agency and ECs by annual safety report.
The NCC is responsible for provide to all the investigators involved in the trial, with appropriate timelines, all the safety information relevant for patient safety.

5. DATA MANAGEMENT

Variables measuring the following items will be gathered: demographics, past and current medical history, clinical and biological breast cancer characteristics, inclusion and exclusion criteria, treatment allocation, schedule, dose reduction, treatment interruption
or permanent withdrawal, concurrent intervention, biochemical examinations, cardiac assessment with ECG and echocardiographic or MUGA measurements, side effects of therapies and adopted interventions, relapses, death, secondary cancer.
CRFs will be provided for the recording of all data. The CRFs will be provided in an electronic form. Trained people at each clinical institution will fill-in the CRFs, according to the data collected on the patient clinical records.
Copies of all clinical examinations, biochemical analyses, ECG and echocardiographic assessments, and of radiological examinations documenting the onset of relapse must be kept by the investigators in the clinical charts.
After filling in, the CRF sheets will be downloaded and printed, signed by the investigator and kept by at his site. The trial monitors will verify that the data filled in the CRF is consistent with those reported on the clinical data charts.

6. STATISTICAL METHODS AND DETERMINATION OF SAMPLE SIZE

6.1 Primary endpoint

The analysis will take the form of a non inferiority test. The sample size calculation is related to the study's primary outcome which is the DFS. Let HR be the ratio between the hazard rate of events following short treatment and the hazard rate of events following long treatment.

Formally, we define the short-treatment to be inferior to long-treatment if the hypothesis that HR ≥ 1.29 is true (null hypothesis), whereas, we define short to be non-inferior to long if HR □ 1.29 (alternative hypothesis. The sample size calculation relates to the study's primary outcome which is the DFS.

The sample size calculation has been amended in respect to the original protocol, due to the following reasons:

a) a slower than expected rate of enrolment has been observed, mainly due to delays attributable to the process necessary to activate centres;

b) less patients than expected were available for randomisation due to the presence of competitive studies in the centres involved in this project, mostly performed by the pharmaceutical industry;

Hence, it has been agreed to conclude the accrual of patients in November 2012. Taking into account the enrolment rate observed in the last months it is reasonable to state that the expect total number of patients enrolled by November 2012 will be 1250.

The study therefore will provide results based on half of the number of patients that was initially established. This change in terms of sample size, provided that alpha is set to 0.05 (one tail), will result in a power of 56%.

All patients will be analysed according both intention-to-treat and per protocol principle. HR for DFS and OS will be estimated according to the Cox model and their relative 90% confidence intervals will also be reported. We will also provide estimates and confidence intervals for the crude hazards and cumulative incidence curves in either treatment arm.

Data analysis will be also carried out by using the Bayesian approach (1). This method will allow us to estimate the posterior probability that the short administration of Trastuzumab is not inferior, in terms of efficacy, to the long administration. The prior distributions will be elicited on the basis of the knowledge available in the literature at the time the analysis is performed. These distributions will be obtained by using the method developed by Parmar (2), whereas the likelihood will be estimated by using the data of the study.

6.2 Secondary endpoints

A site of first treatment failure table will be constructed, summarizing the frequency and crude hazards of recurrence, deaths, events of toxicity. Confidence intervals will be also calculated for relative risks, and cumulative incidences. Groups will be compared using
the log-rank test.

6.3 Interim analysis

The definitive analysis of the primary outcome will take place after 198 events have been reported in the entire population of randomized patients. Interim analyses will be performed annually by the Independent Data Monitoring Committee (IDMC), beginning when 50 events have been reported. The event has been defined as:

1. Death for all cause
2. Local, regional and distant recurrence

3. Contralateral breast cancer, excluding in situ carcinoma
4. Second primary cancer
5. Interruption of Herceptin treatment due to cardiac toxicity

Cardiac events, defined as:

a. CHF with NYHA ≥3
b. Absolute decrease of EF ≥16% from basal values, or absolute decrease ≥ 10% with EF below 50%, or symptomatic cardiac failure, or other cardiac side effects grade 2 or more according to NCI CTCAE (version 3)

No early stopping of accrual is planned in the presence of data supporting rejection of the null hypothesis (that is, if early results support the hypothesis of non-inferiority), but only in the presence of results supporting the null hypothesis (i.e. superiority of the longer regimen). Therefore, interim analyses are to be considered equivalent to standard futility analyses in superiority trials, and no correction of p values for multiple tests is needed.
For interim analyses, the Bayesian monitoring approach developed by Parmar et al will be used. Patients accrual will be stopped as soon as the posterior probability that the null hypothesis is wrong becomes too low when compared with the observed difference in cardiotoxicicty.

6.4 Accrual rate and total sample size

About 100 clinical institutions will participate to the study, referring to 8 Italian Regional Coordinating Centres (RCC), which in turn will be coordinated by one National Coordinating Centre (NCC), responsible for the entire study conduction.
On the basis of the accrual estimated by each Centre and RCC, we plan to enrol 1250 patients up to November 2012.
The study will finish when the 198th event is observed.

6.5 Considerations

It is important to highlight that recently a Finnish study, named SOLD, was registered in the American registry of trials, www.clinicaltrial.gov (NCT00593697). This study assesses similar clinical questions to those handled in the Shorther study. Contact between the two principal investigators has been established. Prof. PierFranco Conte is committed to keeping in touch with the Finnish research group to share information about the studies being performed. Although it has not been formally discussed there is the possibility to eventually carry out a cumulative analysis of data (prospective meta-analysis) relative to common endpoints.

7. STUDY MANAGEMENT

7.1 Monitoring

Start-up visit: after the local ECs approval, a study monitor from the Coordinating Centre will visit the study sites before first study subject entry.
Monitoring during study: the study monitor will visit each centre after the randomization of the first patient, and thereafter will have regular contacts with the investigational sites, including visits to:
- provide information and support to the investigator(s)

- confirm the adherence to the protocol, verify the accurately record of the data in the CRFs, verify the correct investigational product's accountability
- perform source data verification, with direct access to all original records for each subject

The monitor will be available between visits if the investigator(s) or other staff at the centres need information and advice.

7.2 Changes to the protocol

If necessary for the protocol to be amended, the amendment and or a new version the study protocol (Amended Protocol) must be notified and approved by ECs.

8. ETHICS

8.1 Ethics review

The final study protocol, including the final version of the Written Informed Consent Form, must be approved by the ECs. The PI is responsible for informing the ECs of any amendment to the protocol in accordance with local requirements.

8.2 Ethical conduct of the study

The study will be performed in accordance with ethical principles that have their origin in the Declaration of Helsinki and are consistent with ICH/GCP.

8.3 Written informed consent

The PI at each centre will ensure that the subject is given full and adequate oral and written information about the nature, purpose, possible risk and benefit of the study. Subjects must also be notified that they are free to discontinue from the study at any time. The subject's signed and dated informed consent must be obtained prior to conduct any procedure specific for the study. The original signed Written Informed Consent Form must be stored, and a copy must be given to the patient.

8.4 Subject data protection

The Written Informed Consent Form will explain that the study data will be stored in a computer database, maintaining confidentiality in accordance with national data legislation. All computer data will be identified by subject initial and number only.

9. REFERENCES

Slamon DJ, Godolphin W, Jones LA et al. Studies of the HER-2/neu proto-oncogene in human breast and ovarian cancer. Science 1989;244: 707-712.[

Pegram M, Konecny G, O'Callaghan C, et al. Rational Combinations of Trastuzumab With Chemotherapeutic Drugs Used in theTreatment of Breast Cancer J Natl Cancer Inst 2004;96:739-49

Buzdar AU, Valero V, Ibrahim NK, et al. Neoadjuvant therapy with paclitaxel followed by 5-fluorouracil, epirubicin, and cyclophosphamide chemotherapy and concurrent trastuzumab in human epidermal growth factor receptor 2-positive operable breast cancer: an update of the initial randomized study population and data of additional patients treated with the same regimen. ClinCancer Res. 2007 Jan 1;13(1):228-33.

Slamon DJ, Clark GM, Wong SG et al., Human breast cancer: correlation of relapse and survival with amplification of the HER-2/neu oncogene, Science 235 (1987), pp. 177-182.

Slamon DJ, Leyland-Jones B, Shak S et al., Concurrent administration of anti-HER2 monoclonal antibody and first-line chemotherapy for HER2-overexpressing metastatic breast cancer. A phase III, multinational, randomized controlled trial, N Engl J Med 344 (2001), pp. 783-792.

Marty M, Cognetti F, Maraninchi D et al., Randomized phase II trial of the efficacy and safety of trastuzumab combined with docetaxel in patients with human epidermal growth factor receptor 2-positive metastatic breast cancer administered as first-line treatment: the M77001 Study Group, J Clin Oncol 23 (2005), pp. 4265-4274

Piccart-Gebhart MJ, Procter M, Leyland-Jones B et al., Trastuzumab after adjuvant chemotherapy in HER2-positive breast cancer, N Engl J Med 353 (2005), pp. 1659-1672.

Romond EH, Perez EA, Bryant J et al., Trastuzumab plus adjuvant chemotherapy for operable HER2-positive breast cancer, N Engl J Med 353 (2005), pp. 1673-1684

Perez EA, Suman VJ, Davidson N et al. NCCTG N9831. May 2005 update. Slide presentation at the 41st American Society of Clinical Oncology Annual Meeting, Orlando, Florida, May 13-17, 2005. Available at http://www.asco.org/ac/1,1003,_12-002511-00_18-0034-00_19-005815-00_21-001,00.asp. Accessed June 7, 2006.

Slamon D, Eiermann W, Robert N, et al: BCIRG 006: 2nd interim analysis phase III randomized trial comparing doxorubicin and cyclophosphamide followed by docetaxel (AC T) with doxorubicin and cyclophosphamide followed by docetaxel and trastuzumab (AC TH) with docetaxel, carboplatin and trastuzumab (TCH) in Her2neu positive early breast cancer patients. Br Canc Res Treat, 2006, A52.

Joensuu H, Kellokumpu-Lehtinen PL, Bono P et al., Adjuvant docetaxel or vinorelbine with or without trastuzumab for breast cancer, N Engl J Med 354 (2006), pp. 809-820

Sledge GW, O'Neill A, Thor A et al: Adjuvant trastuzumab: long-term results of E2198. Breast Canc Res Treat, 2006, A2075

Tripathy D, Seidman A, Keefe D, et al: Effect of cardiac dysfunction on treatment outcomes in women receiving trastuzumab for HER2-overexpressing metastatic breast cancer. Clin Breast Cancer 5:293-298, 2004

Perez EA, Rodeheffer R: Clinical cardiac tolerability of trastuzumab. J Clin Oncol 22:322-329, 2004

Guarneri V, Lenihan DJ, Valero V et al., Long-term cardiac tolerability of trastuzumab in metastatic breast cancer: the MD Anderson Cancer Center experience, J Clin Oncol 24 (2006), pp. 4107-4115

Tan-Chiu E, Yothers G, Romond E et al., Assessment of cardiac dysfunction in a randomized trial comparing doxorubicin and cyclophosphamide followed by paclitaxel, with or without trastuzumab as adjuvant therapy in node-positive, human epidermal
growth factor receptor 2-overexpressing breast cancer. NSABP B-31, J Clin Oncol 23 (2005), pp. 7811-7819.

Perez EA, Suman VJ, Davidson NE et al. Interim cardiac safety analysis of NCCTG N9831 Intergroup adjuvant trastuzumab trial. J Clin Oncol (Meeting Abstracts) 2005;23:17s

Ewer MS, Vooletich MT, Durand JB, et al: Reversibility of trastuzumab-related cardiotoxicity: New insights based on clinical course and response to medical treatment. J Clin Oncol 23:7820-7826, 2005

Reference Statistical Methods

Spiegelhalter DJ, Myles JP, Jones DR, Abrams KR. Bayesian methods in health technology assessment: a review. Health Technol Assess 2000; 4(38) .

Parmar MKB, Spiegelhalter DJ, Freeman LS. The CHARTS trials: Bayesian design and monitoring in practice. Stat Med 1994; 13:1297-312.
