# Supplementary material for: Tumor-Infiltrating Lymphocytes and Survival Outcomes in Early ERBB2-Positive Breast Cancer: 10-Year Analysis of the ShortHER Randomized Clinical Trial
Source: JAMA Oncol. 2025 Feb 13;11(4):386–93. doi: 10.1001/jamaoncol.2024.6872 (PMC11826437; doi:10.1001/jamaoncol.2024.6872)
Supplement: Supplement 2. — eFigure. Kaplan-Meier overall survival curves comparing patients with high TILs (>20%) vs patients with lower TILs (<20%) in stage-defined groups [file jamaoncol-e246872-s002.pdf]

Supplemental Online Content

Dieci MV, Bisagni G, Bartolini S, et al. Tumor-infiltrating lymphocytes and survival outcomes in early *ERBB2*-positive breast cancer: 10-year analysis of the ShortHER randomized clinical trial. *JAMA Oncol*. Published online February 13, 2025. doi:10.1001/jamaoncol.2024.6872

eFigure. Kaplan-Meier overall survival curves comparing patients with high TILs (>20%) vs patients with lower TILs (<20%) in stage-defined groups

This supplemental material has been provided by the authors to give readers additional information about their work.

© 2025 Dieci MV et al. *JAMA Oncol*.

eFigure. Kaplan-Meier overall survival curves comparing patients with high TILs (≥20%) vs patients with lower TILs (<20%) in stage-defined groups: Stage I (A), Stage II (B), Stage III (C)

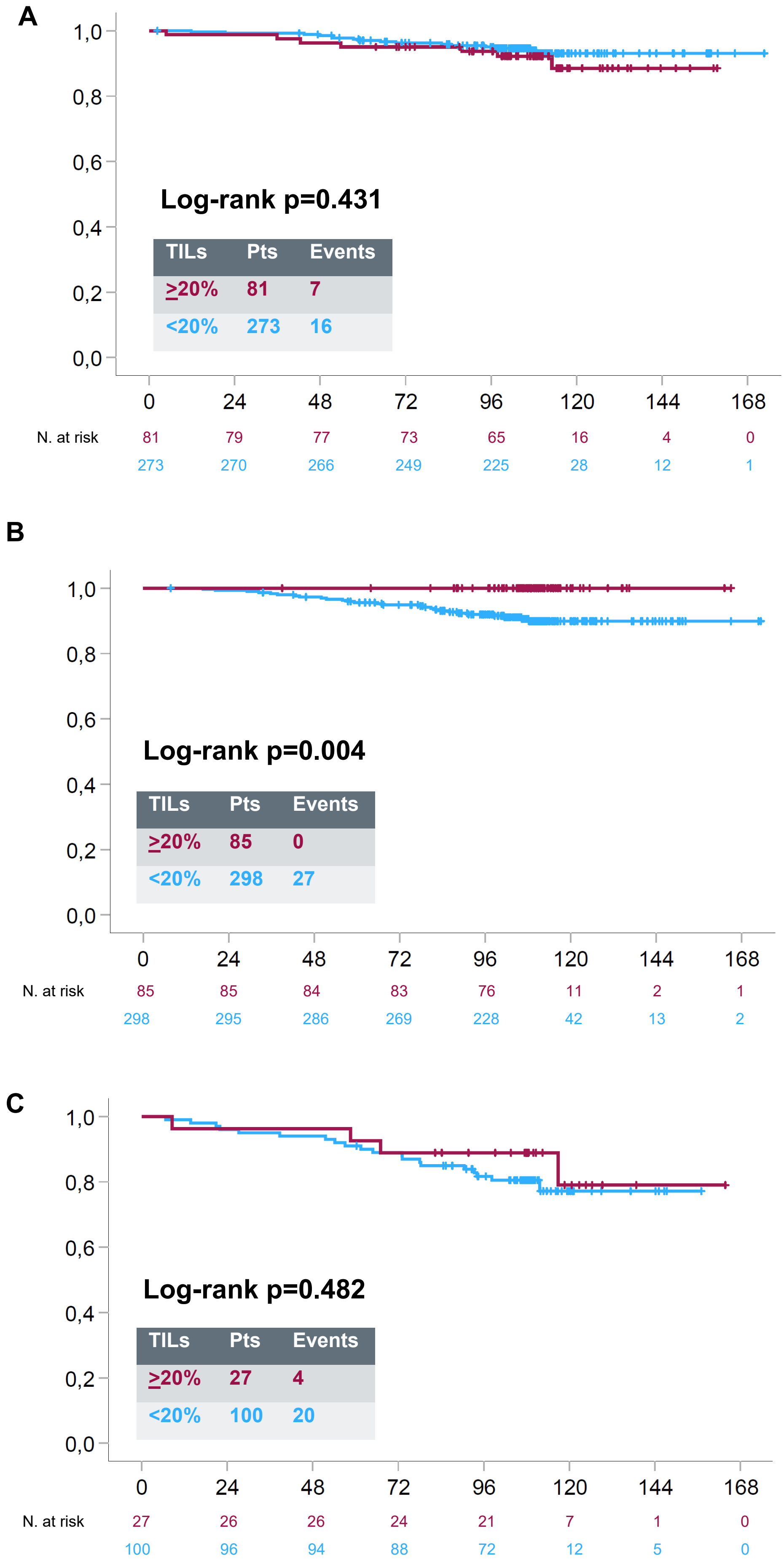

© 2025 Dieci MV et al. *JAMA Oncol.*
