# Supplementary material for: Tumor-Infiltrating Lymphocytes and Survival Outcomes in Early ERBB2-Positive Breast Cancer: 10-Year Analysis of the ShortHER Randomized Clinical Trial
Source: JAMA Oncol. 2025 Feb 13;11(4):386–93. doi: 10.1001/jamaoncol.2024.6872 (PMC11826437; doi:10.1001/jamaoncol.2024.6872)
Supplement: Supplement 3. — Data Sharing Statement [file jamaoncol-e246872-s003.pdf]

Data Sharing Statement

Dieci. Tumor-Infiltrating Lymphocytes and Survival Outcomes in Early ERBB2-Positive Breast Cancer. *JAMA Oncol*. Published February 13, 2025. doi:10.1001/jamaoncol.2024.6872

Data
Additional Information: EUDRACT: 2007-004326-25, ClinicalTrials.gov: NCT00629278
Data available: Yes
Data types: Other (please specify)
Additional Information: Individual patient level data is not publicly available to maintain compliance with trial protocol. Anonymized data are available for non-commercial use from sponsor/principal investigator upon request pending data usage agreement and/or IRB-approved collaboration.
How to access data: Individual patient level data is not publicly available to maintain compliance with trial protocol. Anonymized data are available for non-commercial use from sponsor/principal investigator upon request pending data usage agreement and/or IRB-approved collaboration.
When available: With publication

Supporting Documents
Document types: None

Additional Information
Who can access the data: Individual patient level data is not publicly available to maintain compliance with trial protocol. Anonymized data are available for non-commercial use from sponsor/principal investigator upon request pending data usage agreement and/or IRB-approved collaboration.
Types of analyses: Individual patient level data is not publicly available to maintain compliance with trial protocol. Anonymized data are available for non-commercial use from sponsor/principal investigator upon request pending data usage agreement and/or IRB-approved collaboration.
Mechanisms of data availability: Individual patient level data is not publicly available to maintain compliance with trial protocol. Anonymized data are available for non-commercial use from sponsor/principal investigator upon request pending data usage agreement and/or IRB-approved collaboration.
